# California TRV-based VIGS vectors mediate gene silencing at elevated temperatures but with greater growth stunting

**DOI:** 10.1186/s12870-021-03324-8

**Published:** 2021-11-22

**Authors:** Jamilur Rahman, Ian T. Baldwin, Klaus Gase

**Affiliations:** 1 Department of Molecular Ecology, Max Planck Institute for Chemical Ecology, Hans-Knoell-Str. 8, 07745 Jena, Germany; 2 Present address: Department of Genetics and Plant Breeding, Sher-e-Bangla Agricultural University, Dhaka, 1207 Bangladesh

**Keywords:** *Allene oxide cyclase* (*AOC*), Higher temperature, *Phytoene desaturase* (*PDS*), Tobacco rattle virus (TRV), Virus-induced gene silencing (VIGS), *Nicotiana attenuata*

## Abstract

**Background:**

Tobacco rattle virus (TRV) based virus-induced gene silencing (VIGS), a widely used functional genomics tool, requires growth temperatures typically lower than those of the plant’s native environment. Enabling VIGS under native conditions in the field according to applicable safety regulations could be a revolutionary advance for ecological research.

**Results:**

Here, we report the development of an enhanced thermal tolerant VIGS vector system based on a TRV California isolate. cDNA clones representing the whole viral genome were sequenced and used to construct separate binary plant transformation vectors for functional elements of RNA1 (6765 nt) and RNA2 (3682 nt). VIGS of target genes was induced by transient transformation of the host plant with both vectors or by treating the host plant with sap from already VIGS induced plants. In *Nicotiana attenuata* the silencing efficiency of the *PDS* (*phytoene desaturase*) gene was 90% at 28 °C and 78% at 30 °C. Silencing at these temperatures was more prominent and durable than silencing induced by the widely used TRV PpK20-based pBINTRA6/pTV00 system, but was associated with a viral phenotype. Differences in the suppressor protein and RNA dependent RNA polymerase sequences between the TRV California isolate and PpK20 may be the reason for their different thermal tolerance.

**Conclusions:**

The new TRV California-based VIGS vectors induce gene silencing in *Nicotiana attenuata* at higher temperatures than the existing pBINTRA6/pTV00 vector system, but cause greater growth defects. The new vector system opens up an avenue to study genes functions *in planta* under field conditions.

**Supplementary Information:**

The online version contains supplementary material available at 10.1186/s12870-021-03324-8.

## Background

The manipulation of gene expression represents the gold standard for the proof of gene function in many model organisms. Virus-induced gene silencing (VIGS), a transient form of post-transcriptional gene silencing, has emerged as an extremely powerful functional genomics tool for knocking down the expression of target genes in plants [1–3]. VIGS uses viral vectors harboring a target gene fragment to produce dsRNA which triggers RNA-mediated silencing of the target gene. Transient VIGS is a simple, cost-effective and considerably less time-consuming method to manipulate gene expression for the analysis of gene function than the classical stable transformation [4, 5].

TRV (tobacco rattle virus)-based VIGS has been utilized to effectively manipulate the expression of genes in many important model plant species including Arabidopsis (*Arabidopsis thaliana*), *Nicotiana spp.*, Tomato (*Solanum lycopersicum*), Cotton (*Gossypium arboreum*), *Petunia hybrida*, etc. [6]. TRV-based VIGS has proven the most effective method because TRV has several distinct advantages over other viruses developed for VIGS: relatively mild symptoms of infection, infection of large patches of neighboring cells, migration to growing meristems and thus efficiently into new tissues in all parts of the plant [2, 3, 7].

TRV is a member of the genus *Tobravirus* in the family of *Virgaviridae* and has a bipartite, positive sense single stranded RNA genome (RNA1 and RNA2) [8]. RNA1 and RNA2 are encapsidated separately into tubular, rigid, rod-shaped particles. The RNA1 encoded-proteins are sufficient for replication and movement of the virus within the host plant, while RNA2 encodes proteins for virion formation and nematode-mediated transmission [9]. RNA1 encodes four open reading frames (ORFs) for proteins with predicted molecular weights of 134 and 194 kDa (replicase proteins), 29 kDa (movement protein), and 16 kDa (cysteine-rich protein, a silencing suppressor protein) [10]. RNA1 can replicate and move systemically without RNA2. RNA2 encodes three proteins (coat protein and two non-structural proteins, 29.4 kDa and 32.8 kDa) [11].

Genomic diversity analysis of different TRV isolates revealed that the RNA1 genome is well-conserved among the different strains of TRV. At the nucleotide level, 92–99% similarity is found among different European and American TRV isolates [12]. In contrast to RNA1, RNA2 molecules in various tobravirus isolates show considerable differences in size and composition [13]. RNA2 from different TRV isolates has little nucleotide sequence similarity, for example, TRV Michigan isolate shares only 32–52% identity with European isolates [12]. Thus, the RNA2 genome is highly variable in nature, which results in numerous serotypes and TRV virus strains [14]. This variability of RNA2 might be due to recombination among tobraviruses [9, 15].

A TRV-based VIGS vector system was first described by Ratcliff et al. (2001) [1], afterwards Liu et al. (2002) [16] further modified the vectors. For constructing the first TRV-based VIGS vectors, cDNA from RNA1 of strain PpK20 (*Paratrichodorus pachydermus* Kinshaldy-20) containing the ORFs for the RNA-dependent RNA polymerase (RdR), the 29 kDa and the 16 kDa proteins, was placed between the Cauliflower Mosaic Virus (CaMV) 35S promoter and terminator. cDNA from RNA2 encoding the coat protein was placed between a CaMV 35S promoter and the nopaline synthase terminator. For cloning the target gene sequences a multiple cloning site was introduced downstream from the coat protein gene. The resulting TRV vectors were designated pBINTRA6 and pTV00, respectively [1].

Plant growth conditions such as temperature, relative humidity and age of the inoculated plants, have profound effects on the efficiency and uniformity of VIGS as well as on spread of gene silencing [17]. Among these factors, temperature plays the most critical role and directly influences T-DNA insertion frequency, generation of primary and secondary small interfering RNAs (siRNAs) and spread of the siRNAs to distal organs [18–24]. Depending on plant species, robust TRV-based VIGS occurs at growth temperatures between 19 °C and 25 °C [6, 7]. The optimal growth temperatures for robust silencing after VIGS inoculation are 20–22 °C for Arabidopsis [25], *N*icotiana *benthamiana* [26], *Nicotiana attenuata* [2, 3] and *Nicotiana tabacum* [27], 23–25 °C for cotton [28] and 24 °C for pepper (*Capsicum annuum*) [29].

Experimental analyses in the ecological model plant *N. attenuata*, which originates from the Great Basin Desert in the southwest of the United States, revealed that a growth temperature of 22 °C was optimal to induce the bleaching phenotype by TRV VIGS (pBINTRA6/pTV00 vectors) [2]. Temperatures both lower than 20 °C and greater than 24 °C dramatically reduced the efficiency of silencing (unpublished data). Thus, the available pBINTRA6/pTV00-based TRV-VIGS vectors could not be used as a functional genomics tool for gene silencing in *N. attenuata* grown in the plant’s native environment, where higher temperatures (above 25 °C) commonly occur. Hence, engineering of greater thermal tolerance into the TRV-based VIGS system would be the first and most important step in developing a viable VIGS-based gene silencing system for field work with the *N. attenuata* system. Developing a VIGS vector system that functions under field conditions would be a “game changer” for ecological research. We assumed that the main technical challenge of this TRV-VIGS system was the thermal tolerance of the utilized TRV strain PpK20. The lower temperature requirement of this strain might be attributed to its geographic origin: PpK20 was collected from Kinshaldy, Scotland [30], where the climate is generally cool and annual average temperature is 10.5 °C [31].

Developing a more temperature tolerant TRV VIGS vector system enabling gene silencing in the native environment of *N. attenuata*, taking into account applicable safety regulations, would open up a new avenue for analyzing gene function of this model organism and other tropical or subtropical plant species (e.g., *Solanaceae*, *Cucurbitaceae*, *Poaceae*, etc.) under field conditions. Developing such vectors requires a TRV strain with higher thermal tolerance than the currently used TRV PpK20. We hypothesized that TRV isolates collected from places with higher annual average temperatures than Scotland would allow VIGS to be performed at higher temperatures than those achieved with the current pBINTR6/pTV00 system.

Here we developed a new VIGS vector system using a TRV isolate collected from coastal California (Santa Barbara County), USA, that was designated TRV California. First, we sequenced the complete TRV California genome consisting of RNA1 and RNA2. In the next step, we constructed a new VIGS vector system based on this genome. We noted the sequence differences between the genomes of TRV California and PpK20 and other TRV isolates. The functional analysis of the new TRV-based VIGS system showed that the vectors induced profound gene silencing (up to 90%) at higher growth temperatures (28 °C/30 °C) in *Nicotiana attenuata*, indicating greater thermal tolerance of the new vector system compared to the current vectors, pBINTRA6 and pTV00.

## Results

### Isolation and cloning of the complete genomes of RNA1 and RNA2 of TRV California

**Fig. 1:**
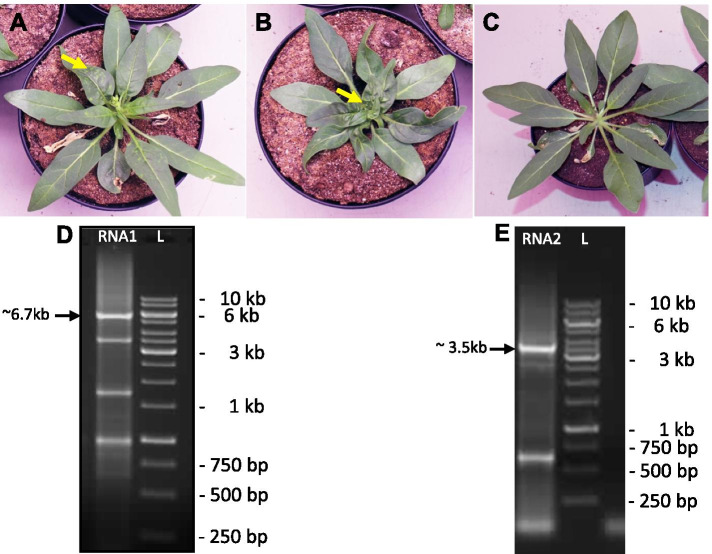
Isolation of the full length cDNA of the TRV California genomes from infected *N. attenuata.* **A** and **B** Inoculated plants show the viral symptoms e.g. mottling, localized necrotic lesions, chlorotic spots on the leaves and leaf-deformities in the TRV California inoculated plants. **C** No such symptoms appeared in the control plants, where only buffer was inoculated. Photographs were taken at 15 dpi. The yellow arrows indicate the virus symptoms on leaves. **D** and **E** 1% agarose gel images of PCR amplification of cDNA from full length RNA1 (D, lane RNA1, size ~ 6.6 kb) and RNA2 (E, lane RNA2, ~ 3.5 kb) with TRV California genome specific primers. Lanes ‘L’ represent the 1 kb DNA ladder. The gel images are cropped

We amplified the virus from the infected spinach leaf samples [32] by inoculation of the leaves of four host plant species viz. spinach, pepper, *N. benthamiana and N. attenuata.* One week after inoculation typical TRV infection symptoms, e.g. blotchy light and dark discoloration of leaf tissue, chlorotic spots and localized necrotic lesions appeared on the leaves of host plants (Figs. 1A, B and S1). To confirm the TRV infection of these plants, we amplified and sequenced diagnostic PCR-fragments of RNA1 cDNA with sizes of 341 bp (Fig. S2A) and 611 bp (Fig. S2B) using TRV RNA1 genome specific primers. A comparison of the obtained sequence with TRV sequences in GenBank confirmed their identity as TRV fragments.

To obtain the whole genome sequences, the full length RNA1 and RNA2 genomes of TRV California strain were reverse transcribed and amplified by PCR using the respective genome specific primers and total RNA from TRV California infected plant samples (Figs. 1D and E). The amplified full-length RNA2 genome was cloned in pCR Blunt II-TOPO. Since a TRV RNA1 protein encoded in the RdR open reading frame (ORF) is toxic to *E. coli* [1], it was not possible to clone the full-length cDNA of this RNA. To overcome this problem, we divided the TRV RNA1 cDNA into three fragments, thereby interrupting the RdR ORF. The three fragments (2.1 kb, 3.4 kb and 1.2 kb) were PCR amplified and cloned separately in pJET1.2.

We sequenced the full-length TRV California RNA1 and RNA2 genomes from the cloned TRV fragments. The genome organization of TRV California RNA1 and RNA2 was similar to RNA1 and RNA2 described for other TRV isolates [9, 33, 34].

**Fig. 2:**
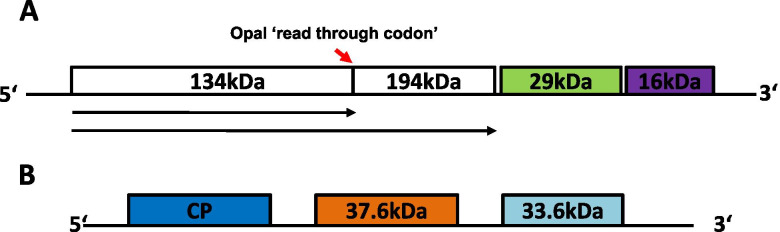
Schematic representation of the genomic organization of RNA1 and RNA2 of the TRV California isolate. OFRs are boxed and protein sizes are denoted. Panel **A** represents RNA1 genome encoding three proteins, the 194 kDa (read through 134 kDa) RDR protein, 29 kDa movement protein and 16 kDa silencing suppressor protein. Panel **B** shows the RNA2 genome encoding the coat protein (CP) and the 37.6 kDa and 33.6 kDa proteins

RNA1 consists of 6765 nt and encodes four putative ORFs of proteins with molecular weights of 134 kDa, 194 kDa, 29 kDa, and 16 kDa and 5′ and 3′ non-coding regions (NCR) of 179 nt and 252 nt, respectively (Fig. 2A). The ORF designation follows that in MacFarlane (1999) [9] and Crosslin et al. (2010) [12]. The first ORF starts at position 180 and ends with an UGA (opal) stop codon at position 3741 nt (Fig. 2A). It encodes a 134 kDa protein. Read through of this stop codon continues the ORF for further 1557 nt (519 amino acid residues) to encode the 194 kDa protein, the RdR. The 134 kDa protein comprises the amino terminal portion of the 194 kDa RdR and contains the helicase and nucleotide-binding motifs. The 29 kDa ORF encodes a putative movement protein [12]. The 16 kDa protein is a small, cysteine-rich RNAi silencing suppressor protein [10].

RNA2 consists of 3682 nt and contains three putative ORFs and 5′ and 3′ NCRs of 522 nt and 568 nt, respectively (Fig. 2B). The first ORF encodes the 22.4 kDa putative coat protein of the virus. The second ORF encodes a 37.6 kDa protein and the third ORF codes for a protein of 33.6 kDa (Fig. 2B). Lastly, the 3′ end terminal of RNA2 contains a region that is proposed to originate from the 3′ end of RNA1 of TRV [35].

### Phylogenetic analysis of TRV California and PpK20

To compare the sequence differences in the genomes of TRV California, PpK20 and other TRV isolates, we performed pairwise alignment analysis and constructed phylogenetic trees using the RNA1 and RNA2 genomes of PpK20 and other TRV strains submitted as full-length genomes to the NCBI data base.

**Fig. 3:**
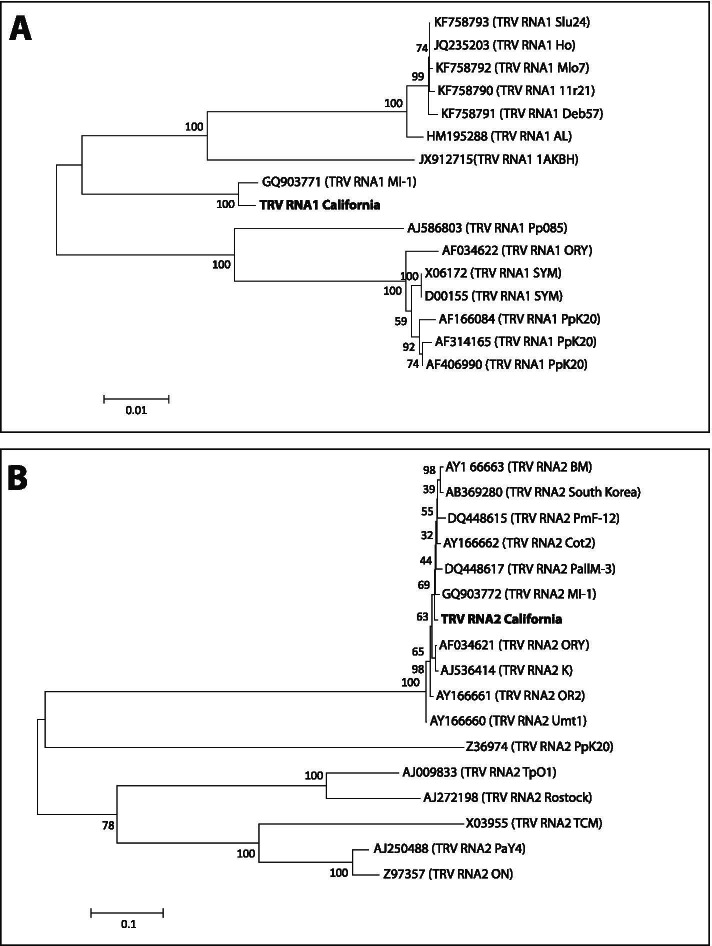
Phylogenetic analysis of RNA1 and RNA2 genomes of the TRV California isolate. Phylogenetic trees showing the relationship between A) RNA1 genomes of fifteen TRV isolates and RNA1 of the California isolate and B) between RNA2 genomes of sixteen TRV isolates and RNA2 of the California isolate. The nucleotide sequences were aligned using the ClustalW method and the trees were constructed by neighbor-joining method using MEGA version 5 [36]. Numbers close to the nodes indicate bootstrap values and the scale shows 0.1 nucleotide substitutions per site. The deduced nucleotide sequences were retrieved from the NCBI GenBank database

The phylogenetic analysis of different RNA1 genomes revealed that the TRV California isolate is positioned in a clade of American isolates separate from European TRV isolates e.g., PpK20 (Scotland), PpO85 (Netherland) (Fig. 3A). The tree shows that the PpK20 isolate, which shares 92.5% sequence identity with the TRV California strain, is positioned in a clade of out-groups separate from the TRV California isolate (Fig. 3A), suggesting that the isolates are distantly related to each other. However, TRV isolates e.g. Deb57, Mlo7, 11r21, Deb57, Slu24 originating from Poland and isolate Ho originating from Germany were in the same clade as the TRV California strain, but in different clusters (Fig. 3A).

The phylogenetic tree of the RNA2 genomes showed that the American isolates, BM, PmF-12, Cot2, Pallm-3, M1, ORY, OR2 and Umt1 are clustered in the same clade as the TRV California isolate indicating their parallel evolutionary pattern (Fig. 3B). The European isolates, except the PpK20, are grouped in clades different from the TRV California isolate and form different groups in the phylogeny tree (Fig. 3B). The PpK20 strain, which shares 95% sequence identity with the TRV California strain, was positioned in the same clade as the California isolate, albeit in a different branch of the clade (Fig. 3B).

### Construction of a new VIGS vectors system based on TRV California

**Fig. 4:**
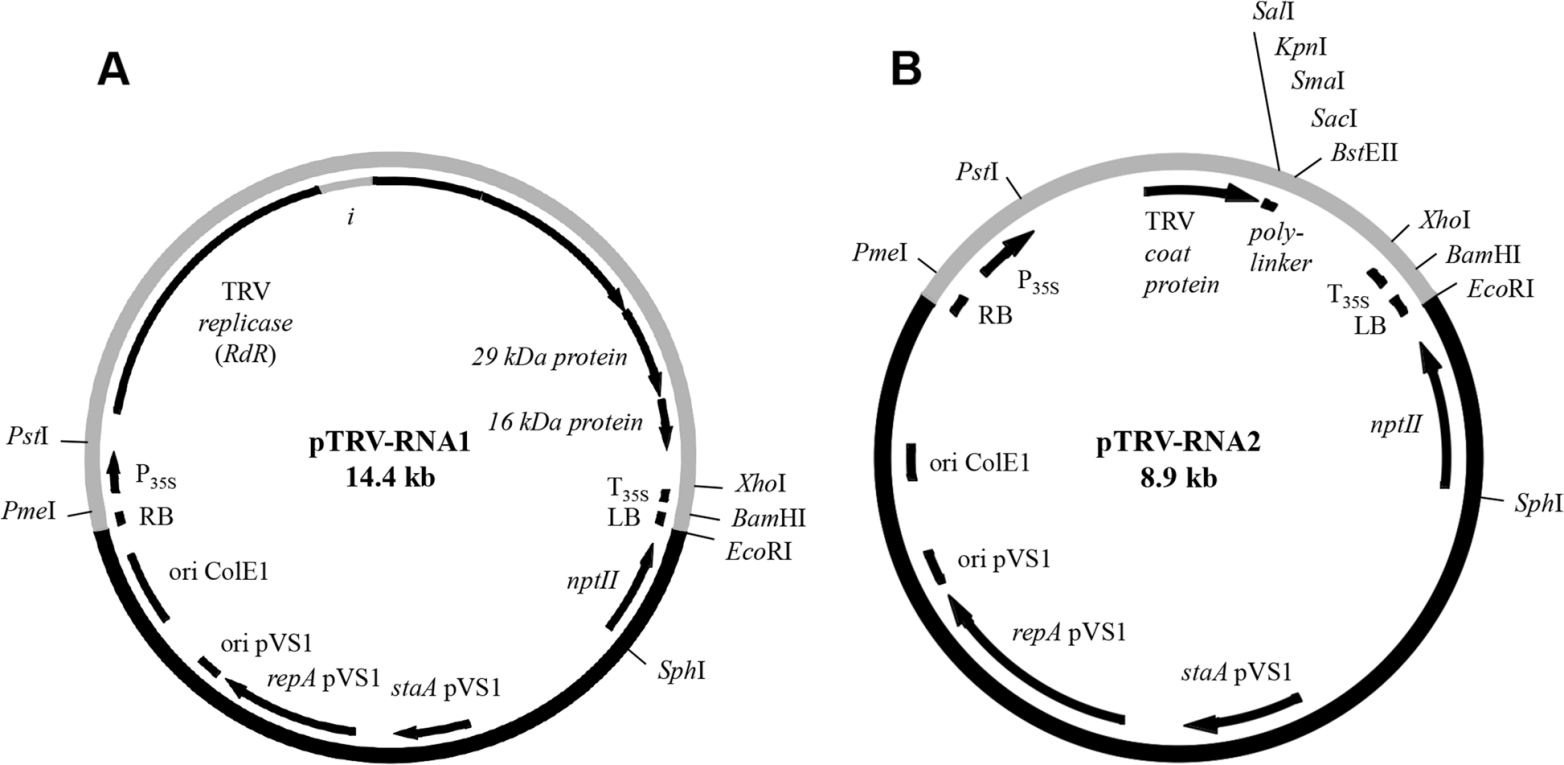
pTRV-RNA1 and pTRV-RNA2 VIGS vectors based on the TRV California isolate. **A** pTRV-RNA1 carrying the full length TRV California RNA1 interrupted by an intron on its T-DNA. **B** pTRV-RNA2 carrying the coat protein and the 3′ and 5′ regions from TRV California RNA2. The gray sections of the circles represent the T-DNA, and the black sections represent the vector backbone originating from the binary plant transformation vector pSOL9DEF1. Abbreviations: i, intron 3 of the *A. thaliana NIA1* gene for *nitrate reductase* (Z19050); LB, left border; *ntpII, neomycin phosphotransferase II*; ori ColE1, origin of replication of ColE1; ori pVS1, origin of replication of pVS1; P_35S_, 35S promoter from cauliflower mosaic virus; RB, right border; RdR, RNA-dependent RNA polymerase; repA pVS1, replication protein from pVS1; staA pVS1, plasmid stability protein from pVS1; T_35S_, 35S terminator from cauliflower mosaic virus

To construct the TRV California based VIGS vector system we mainly followed the design of the previously reported pBINTRA6 and pTRV00 vectors based on the TRV PpK20 isolate [1]. We created two separate binary plant transformation vectors for the cDNA of TRV California RNA1 and RNA2 based on pSOL9DEF1 (pTRV-RNA1 and pTRV-RNA2, Fig. 4A and B). The pSOL9 vector backbone has been successfully used before for the stable transformation of *N. attenuata* and carries outside of the T-DNA only the functional elements required for bacterial replication [37].

The pTRV-RNA1 (14.4 kb) construct contains a full-length infectious cDNA of California TRV RNA1. The RdR ORF has been interrupted by the insertion of intron 3 of the *A. thaliana NIA1* gene for *nitrate reductase* (GenBank Z19050) to prevent the expression of a protein that is toxic to *E. coli* [1]. The construct also contains the full length ORFs of the 29 kDa protein (movement protein) and the 16 kDa protein (silencing suppressor protein) of the TRV California strain (Fig. 4A).

In the pTRV-RNA2 (8.9 kb) construct, the non-essential 37.6 kDa and 33.5 kDa protein genes were removed, leaving only the 5′ and 3′ untranslated regions and the coat protein (CP) gene of the virus (Fig. 4B). A polylinker has been introduced directly downstream from the coat protein ORF, facilitating the cloning of the target gene fragments.

### pTRV-RNA1 and pTRV-RNA2 induce gene silencing under higher temperature growth conditions (26–30 °C)

To test whether the newly developed pTRV-RNA1 and pTRV-RNA2 vectors can be used for VIGS under field conditions, we determined the upper temperature limits of their silencing function. For this, we constructed pTRV2:PDS by cloning a 206 bp fragment of the *phytoene desaturase (PDS)* gene from *N. benthamiana* in the polylinker of pTRV-RNA2 and again studied bleaching due to *PDS* silencing in *N. attenuata*.

**Fig. 5:**
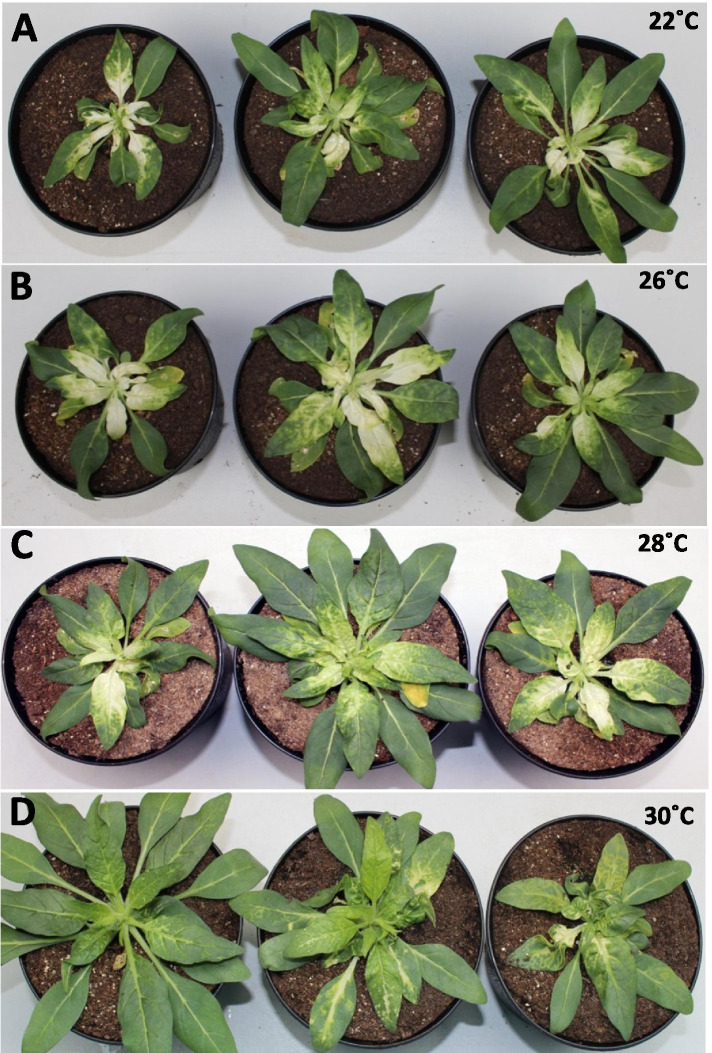
TRV California vectors induce *PDS* silencing in *N. attenuata*. Bleaching phenotype of *N. attenuata* after *PDS* silencing induced by pTRV-RNA1 and pTRV-RNA2:PDS at 22 °C, 26 °C, 28 °C and 30 °C growth temperature. The photographs of the plants were taken at 13 dpi (days post inoculation) for 22 °C, 26 °C, 28 °C (panels **A**, **B**, **C**) and at 17 dpi for 30 °C (panel **D**)

**Fig. 6:**
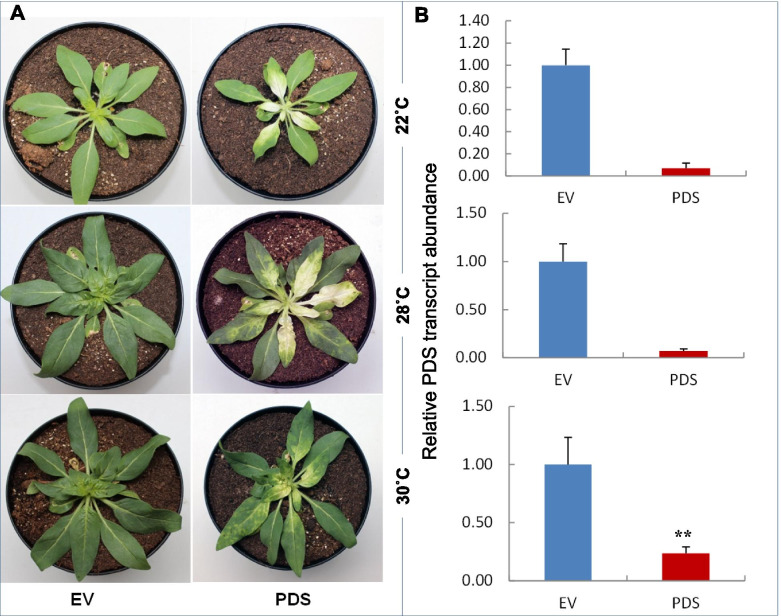
TRV California vectors induce *PDS* silencing at different growth temperatures in *N. attenuata*. **A** Plants inoculated with pTRV-RNA1/pTRV-RNA2:PDS (PDS) expressed *PDS* silencing bleaching phenotype at 22 °C, 28 °C and 30 °C conditions. Plants inoculated with the empty vector control (EV) pTRV-RNA1 + pTRV-RNA2:EV (EV) did not have the bleaching phenotype, but displayed viral symptoms in the inoculated leaves at the different temperatures. **B** Relative PDS transcript abundance in pTRV-RNA1/pTRV-RNA2:PDS (PDS) and pTRV-RNA1 + pTRV-RNA2:EV (EV) inoculated plants grown at 22 °C, 28 °C and 30 °C. The strongest PDS silencing was observed in plants maintained at 28 °C (both with respect to bleaching phenotype and relative PDS transcript abundance). The data of panel B are shown as mean values ±SD; F-test, ** *P* < 0.01. Photographs of the inoculated plants and the samples for RNA isolation were taken at 13 dpi (days to post inoculation)

Initially, we used a growth temperature of 22 °C, as this is the optimal temperature to perform VIGS with the PpK20 derived vectors pBINTRA6 and pTRV00 [3]. The pTRV-RNA1 and pTRV-RNA2:PDS inoculated *N. attenuata* plants showed the bleaching phenotype in the newly emerged leaves at 22 °C (Figs. 5A and 6A). Bleaching started after 10 dpi and continued until seed set of plants. The quantitative PCR data indicated that 90% of the *PDS* transcripts were silenced in the TRV California VIGS inoculated plants compared to the empty vector (EV) pTRV-RNA2 inoculated control plants (Fig. 6B) at 22 °C. This result indicates that the TRV California derived VIGS vectors are functional when *N. attenuata* is grown at 22 °C. Morphologically, all pTRV-RNA2:PDS and EV inoculated plants were stunted with slow growth compared to non-inoculated WT type plants (Fig. S3).

In the following experiment with vectors pTRV-RNA1 and pTRV-RNA2:PDS, VIGS inoculated *N. attenuata* plants, which were maintained and also inoculated at 22 °C growth conditions, were moved to different higher temperature conditions, e.g., 26 °C, 28 °C, 30 °C, 32 °C. At 13 dpi the bleaching phenotype appeared in the newly emerged leaves of inoculated plants kept at 26 °C, 28 °C, 30 °C (Figs. 5B-D and S3B-D), but developed into a full bleaching phenotype only in the young emerged leaves of plants maintained at 26 °C and 28 °C. The bleaching of plants kept at 30 °C was scattered and concentrated in leaf veins (Fig. 5D and S3D). We repeated the experiments at least three times and obtained similar results (Table S3). About 77 and 73% of the inoculated plants showed the bleaching phenotype at 26 °C and 28 °C, respectively, while only 70 and 40% of the inoculated plants had bleaching symptoms at 22 °C and 30 °C, respectively (Table S3). Thus, the results revealed that TRV California derived VIGS vectors induced stronger *PDS* silencing at 26 °C and 28 °C than at 22 °C and 30 °C.

To quantify the *PDS* silencing in the inoculated plants, we measured the relative abundance of *PDS* transcripts in VIGS silenced and EV control plants. We found that 93% of the *PDS* transcripts were silenced at 28 °C, while 76% of the *PDS* transcripts were silenced at 30 °C (Fig. 6B). Neither bleaching phenotype nor virus symptoms were found in inoculated plants kept at 32 °C or higher temperatures (data not shown).

### Comparison of silencing efficiency of TRV California and PpK20 derived vectors under higher temperature growth conditions (28–30 °C)

**Fig. 7:**
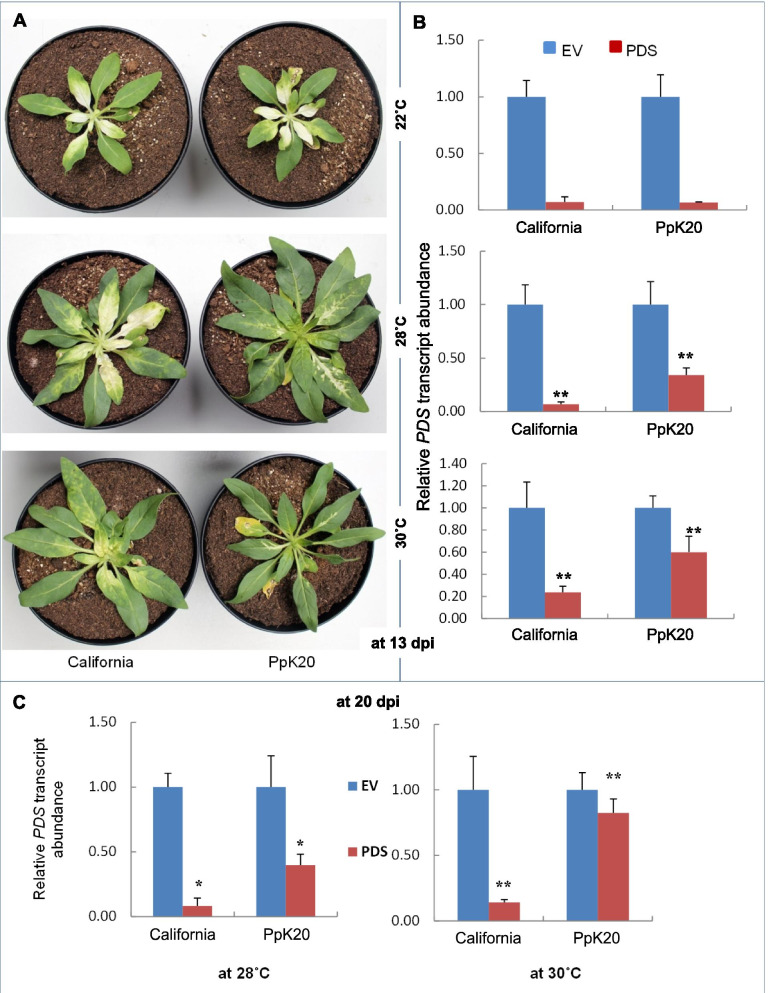
Phytoene desaturase (*PDS*) silencing efficiency in *N. attenuata* at different growth temperatures. **A** Bleaching phenotype of plants inoculated with TRV California (pTRV-RNA1/pTRV-RNA2:PDS; California) and PpK20 (pBINTRA6/pTVPD; PpK20) based VIGS vectors for *PDS* silencing. **B** Relative *PDS* transcript abundance in the same plants compared to empty vector controls (EV). Growth temperatures after inoculation (22 °C, 28 °C and 30 °C) are indicated. Pictures and samples for *PDS* transcript quantification were taken at 13 dpi (days post inoculation). **C** Relative *PDS* transcript abundance of plants inoculated with sap from *N. attenuata* pre-inoculated with the same plasmid combinations as above. Growth temperatures after sap-inoculation (28 °C and 30 °C) are indicated. Samples for *PDS* transcript quantification were taken from apical leaves at 20 dpi. Transcript abundance in B) and C) is shown as mean values ±SD (Student’s t-test, * *P* < 0.05 and ** *P* < 0.01)

We directly compared the *PDS* silencing efficiency of the TRV-California (pTRV-RNA1/pTRV-RNA2:PDS) and PpK20 (pBINTRA6/pTVPD) vector systems under relatively high temperature conditions*.* Both pTRV-RNA2:PDS and pTVPD carry the same 206 bp target fragment of the *phytoene desaturase (PDS)* gene from *N. benthamiana* in antisence orientation. Silencing efficiency was always determined relative to the respective EV (pTRV-RNA2 and pTV00) control plants. After inoculation, *N. attenuata* plants were kept at different temperatures, e.g. from 22 °C to 30 °C, in growth chambers. We found almost similar *PDS* silencing efficiencies for both vector systems at a growth temperature of 22 °C, as evident by a similar type of photo bleaching phenotype observed in the leaves of the inoculated plants (Fig. 7A). Transcript quantification showed that at 22 °C almost 90% of the *PDS* transcripts were silenced by both vector systems (Fig. 7B). This result indicates that both vectors systems induce profound gene silencing at 22 °C. We observed that *PDS* gene silencing induced by the PpK20 vector system in *N. attenuata* was reduced under higher temperature conditions, only 67 and 40% of the *PDS* transcripts were silenced at 28 °C and 30°, respectively (Fig. 7A and B). In contrast, the TRV California derived VIGS vectors yielded almost 90 and 79% silencing of the *PDS* transcripts at 28 °C and 30 °C, respectively (Fig. 7A and B). The results clearly indicate that the TRV California derived VIGS vector system is more thermal tolerant than the PpK20 derived vector system.

To further examine systemic gene silencing in the newly grown apical leaves of *N. attenuata*, we inoculated plants with the sap of pre-established plants silenced by TRV California and PpK20 vectors and allowed them to grow further until 20 dpi. We found that the bleaching phenotype was spreading systemically in the newly emerged apical leaves of TRV California vector sap-inoculated plants kept at 28 °C and 30 °C (Fig. S4A and B). In plants inoculated with PpK20 vector sap and kept under the same temperature conditions, we observed a weak bleaching phenotype concentrated only in the vein regions of basal leaves (Fig. S4A and B). qPCR quantifications revealed that almost 90 and 84% of the *PDS* transcripts were silenced in the TRV California vector sap treated plants, while only 60 and 20% of the *PDS* transcripts were silenced in the PpK20 vector sap treated plants kept at 28 °C and 30 °C, respectively (Fig. 7C).

### RNA1 and RNA2 vector swap experiments reveal the significance of TRV RNA1 for temperature tolerance

The TRV California based VIGS vector system is functional at higher temperatures than that based on PpK20. To figure out whether the RNA1 or RNA2 genomes mainly determine the thermal tolerance of both TRV isolates, we conducted RNA1 and RNA2 vector swap experiments in *N. attenuata* and quantified bleaching due to *PDS* silencing with the mixed PpK20/California TRV vectors (Fig. S5). For *PDS* silencing, RNA1 vectors pBINTRA6 and pTRV-RNA1 and RNA2 vectors pTVPD and pTRV-RNA2:PDS were used. We found that combining the RNA1 and RNA2 genomes from the two different TRV isolates still results in functional viruses. The vector combination pTRV-RNA1 (TRV California) and pTVPD (PpK20) induced *PDS* silencing at both 22 °C and 28 °C, while the vector combination pBINTR6 (PpK20) and pTRV-RNA2:PDS (TRV California) induced *PDS* silencing only at 22 °C, but not at 28 °C. No bleaching phenotype was observed at 30 °C in both combinations of the vectors (Fig. S5). Since the TRV California VIGS vectors are still functional at 30 °C, these results suggest, that mainly RNA1 determines the higher temperature tolerance of this isolate, but that RNA2 also contributes to it.

### Silencing of the ecologically relevant *allene oxide cyclase* (*AOC*) gene in *N. attenuata* by the TRV California vectors at 28 °C and 30 °C

**Fig. 8:**
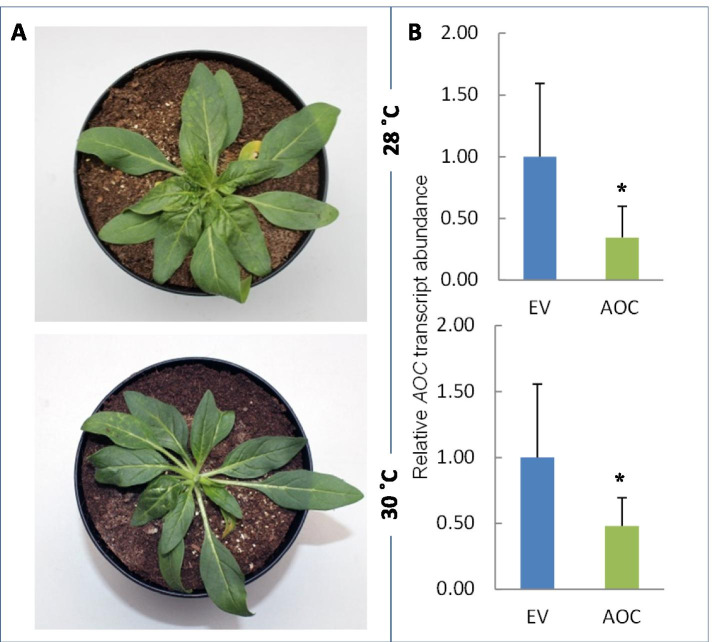
Allene Oxide Cyclase (*AOC*) silencing induced by TRV California based VIGS vectors in *N. attenuata*. **A** Plants inoculated with TRV California based VIGS vectors (pTRV-RNA1/pTRV-RNA2:AOC) for *AOC* silencing. Pictures were taken at 13 dpi (days post inoculation). **B** Relative *AOC* transcript abundance in the same plants compared to empty vector controls. Growth temperatures after inoculation (28 °C and 30 °C) are indicated. Apical leave samples for *AOC* transcript quantification were taken at 13 dpi and 5 h after treatment of the plants with *Manduca sexta* oral secretion (OS). Transcript abundance is shown as mean values ±SD (Student’s t-test, * *P* < 0.05)

We used the TRV California vector system to silence the ecologically relevant *allene oxide cyclase* (*AOC*) gene at higher temperatures in *N. attenuata*. The *AOC* gene was chosen as it is a single copy gene in the genome of *N. attenuata* and catalyses an essential step in the biosynthesis of the phytohormone jasmonic acid (JA). Silencing *AOC* blocks the biosynthesis of JA and its derivatives. Jasmonates are important signals in plant stress responses and plant development [38] and mediate many important ecological responses [39]. The expression of the *AOC* gene is up-regulated after wounding both locally and systemically and after treatment with oral secretion (OS) of herbivores [40]. To silence the *AOC* gene in *N. attenuata*, we co-inoculated WT plants with pTRV-RNA1 and pTRV-RNA2:AOC or pTRV-RNA1 and pTRV-RNA2 (EV control) and grew them at 28 °C and 30 °C. To induce the *AOC* gene, the inoculated plants were treated with OS of *Manduca sexta* larvae at 13 dpi. Leaf samples for total RNA isolation were harvested 5 h after OS induction. qPCR with the *AOC* transcripts from the treated plants maintained at 28 °C and 30 °C revealed that 65 and 52%, respectively, of the *AOC* transcripts were silenced by pTRV-RNA2:AOC relative to the EV control (Fig. 8A and B). Morphologically, the AOC silenced plants had feeble phenotypes compared to the EV inoculated plants.

## Discussion

TRV-based VIGS is very popular as a successful gene knock-down technique. However, the thermal tolerance limit of the current vectors remains the main impediment for its application in diverse plant species and at temperatures above 22 °C as well as under field conditions. Hence, developing a thermal tolerant TRV based VIGS vector could be valuable for plant species that grow in tropical and sub-tropical habitats and plants growing under climate change conditions. Here, we report a new vector system based on the TRV California isolate that induces gene silencing in *N. attenuata* at temperatures of up to 30 °C*.*

The origin and genome structure of different TRV strains are quite divergent. TRV virus is ubiquitous, found in Asia [41], Africa [42], Europe [30, 43] and America [12], demonstrating its wide range of adaptability. TRV has a bipartite RNA genome. In contrast to the relatively conserved genome organization of RNA1, the corresponding RNA2 genomes of different TRV isolates differ in length, genome organization and the RNA1-related recombinant region [43]. Hernandez et al. (1996) [44] reported that the RNA2 composition of a tobravirus (TRV isolate PpK20) may change rapidly during serial passages through the host tobacco plant, while seven different RNA2 species were found to be associated with one RNA1 species in TRV infected *Alstroemeria* plants [13]. Here, the pair-wise alignment and phylogeny analysis of RNA1 genomes showed that the TRV California isolate has 93–94% identity with other TRV isolates reported from different regions of the world (Fig. 3A). The phylogeny analysis shows that the European TRV isolate PpK20 is positioned in a separate clade of out-groups from the California isolates suggesting these isolates were evolved differently and are quite diverse (Fig. 3A). The RNA2 genome of TRV California isolate showed 97–99% identity with RNA2 genomes of American and Asian TRV isolates, while the genome had 90–94% identity with RNA2 genomes the European TRV isolates indicating genomic diversity among RNA2 genomes (Fig. 3B). This result is consistent with previous reports that the RNA2 genome varies considerably among TRV isolates [9, 45]. 99% identity regarding genomic structures of both RNA1 and RNA2 is found between TRV California and a Michigan TRV, both isolates from the USA. These two isolates are placed in same clade of the phylogenic tree, suggesting these American isolates might have evolved from the same ancestors (Fig. 3A).

Among the different types of VIGS vectors, TRV-based vectors have proven the most effective ones because TRV has several distinct advantages over other viruses used for VIGS experimentations. The first TRV based VIGS vectors were developed by Ratcliff et al. (2001) [1], later on the vector system was modified by Liu et al. (2002) [16]. Both the vector systems are based on the TRV PpK20 isolate. Our new vectors based on the TRV California isolate differ from the previously reported pBINTRA6 and pTV00 vectors [1] in the sequences of the two viral genomes and in vector backbones. The pBINTRA6 vector based on the PpK20 isolate offers many advantages, but relatively low growth temperatures (20 °C to 22 °C) are required for efficient and robust silencing [16]. Higher temperatures of 25 °C and more completely prevent the formation of infectious virus particles. Therefore, in the pBINTRA6/pTV00 system at these temperatures gene silencing is abrogated in *N. attenuata*. In contrast, the new vectors based on TRV California successfully induce the gene silencing at higher growth temperatures (up to 30 °C) in *N. attenuata* (Figs. 5, 6 and 7). At a lower growth temperature (22 °C), both the PpK20 and TRV California derived vector systems induced profound *PDS* silencing (Fig. 7). Increasing the growth temperature results in a strong reduction of silencing induced by the PpK20 system in *N. attenuata*, the *PDS* silencing efficiency is only about 67 and 40% at 28 °C and 30 °C, respectively. In contrast, in plants inoculated with the TRV California vectors we found almost 90 and 79% *PDS* silencing at 28 °C and 30 °C, respectively (Fig. 7A and B). In addition, the systemic spread of TRV California vector induced gene silencing reached into the newly emerged apical leaves at 28 °C and 30 °C, while only very faint bleaching in the vein regions of older basal leaves was observed in plants inoculated with the PpK20 derived vectors (Fig. S4). From these results, we infer that the TRV California vectors are capable of inducing persistent gene silencing at growth temperatures up to 30 °C in *N. attenuata*.

Our underlying assumption for developing a higher temperature tolerant VIGS vector system was that TRV isolates collected from places with higher annual average temperatures would allow VIGS at higher temperatures than those collected from lower temperature regions. The TRV PpK20 isolate, which is the origin of the viral elements on pBINTRA6 and pTV00, was collected from the soil of a potato farm in Kinshaldy, Scotland [30, 46] whereas the TRV California strain was collected from commercially grown spinach in coastal California (Santa Barbara County) [32]. Meteorological information on average temperatures of the geographic origins of these two TRV isolates reveals the expected differences. For example, the monthly average temperatures of Santa Barbara, CA, USA varies from 13 °C in January to 22 °C in September (Table S4), while the monthly average temperature of Scotland varies from 5 °C in January to 16.6 °C in August (Table S5). Thus, the different acclimatized temperatures of these two TRV isolates are consistent with the improved thermal tolerance of California VIGS vectors. However, this greater apparent thermal tolerance is associated with more severe growth stunting. Morphologically, the TRV California vectors inoculated plants showed greater growth retardation and feebler phenotypes than the plants inoculated with the PpK20 vectors (Fig. S4). These stronger viral phenotypes could be due to stronger virulence and pathogenicity traits. Genes of interest with expected phenotypes similar to these stronger viral phenotypes can clearly not be used for VIGS experiments with the TRV California vectors. The RNA1 and RNA2 vector swap experiments between the PpK20 and TRV California genomes (Fig. S5) suggested that mainly RNA1 determines the higher thermal tolerance of the TRV California vectors, but also RNA2 contributes to it.

The RNA1 genome of TRV contains the RdR polymerase, movement protein and suppressor protein. The virulence and pathogenicity of viruses are generally determined by RNAi suppressor proteins of viruses [47–49]. The suppressor proteins suppress the host RNA silencing defence mechanism and thus help the viruses to replicate and spread in their hosts [49–52]. We found significant differences between the sequences of the 16 kDa suppressor proteins of the TRV California and PpK20 isolates. The pairwise alignment of both 16 kDa suppressor proteins shows about 13% differences, while 1.7 and 2.4% variations are found between the two RdR polymerases and movement proteins, respectively (Figs. S6 and S7). We found about 44% differences between the coat proteins of TRV California and PpK20 (Fig. S7). Perhaps, the sequences differences in 16 kDa, RdR polymerase, movement proteins and coat proteins that evolved in TRV California are responsible for the higher virulence, but also for the higher and thermal tolerance of this virus.

## Conclusions

The new VIGS vector system based on the TRV California isolate induces gene silencing in *N. attenuata* at temperatures of up to 30 °C*.* Gene silencing induced by TRV California vectors at these temperatures was more prominent and durable than silencing induced by the current TRV vector pBINTRA6/pTV00 system, suggesting that the new vector system is more thermal tolerant, but was associated with more pronounced growth defects. The greater thermal tolerance of the new vector system might be due to the sequence variations in suppressor protein, RdR polymerase and movement protein. The new vector system opens up an avenue to study genes functions *in planta* under field conditions and could be a revolutionary advance for ecological research.

## Methods

### Tobacco rattle virus California strain

The TRV California strain was collected by Hsing-Yeh Liu from commercially grown spinach (*Spinacia oleracea*) in coastal California (Santa Barbara County), California, USA [32]. The collected virus was then identified as TRV by electron microscopy and serological and molecular analyses [32]. The formal identification of the plant material used in our study was undertaken by Steven T. Koike, Tongyan Tian, and Hsing-Yeh Liu [32]. Spinach leaf samples infected with this virus were kindly provided by Dr. Bill Wintermantel, USDA-ARS, Salinas, CA, USA. No voucher specimen of this material has been deposited in a publicly available herbarium, but − 80 °C frozen virus infected spinach leaf samples from this material are available upon request.

### Plant material and growth conditions

The 31st inbred generation of the *N. attenuata* “Utah” ecotype, originally collected from a native population at a field site located in Utah (USA) was used in the experiments. Additionally, for the amplification of the TRV California isolate, healthy seedlings of spinach, pepper and *N. benthamiana* were used. Wild-type seeds of *N. attenuata* were surface sterilized and germinated on Gamborg’s B5 medium (Duchefa, www.duchefa-biochemie.com) as previously described [53]. After 10 days the seedlings were carefully removed from the agar and transferred to soil in small Teku plastic pots (www.poeppelmann.com) in the glasshouse. Again, after 10 days in Teku pots, seedlings were transferred to 1 L pots in soil in a growth chamber. Growth chamber conditions were temperatures of 22 °C, 26 °C, 28 °C, 30 °C and 32 °C, relative humidity of 65% and the duration of 16/8 h of day and night cycle and light intensity of 135–195 μMols^− 1^ m^− 2^ PAR.

### Bacterial strains, TRV PpK20 based vectors, enzymes and oligonucleotides

For cloning and vector construction, chemically competent cells of *E. coli* TOP10 (Invitrogen, www.thermofisher.com), and for plant transformation *Agrobacterium tumefaciens* GV3101 [containing plasmids pMP90 (pTiC58DT-DNA) and (as helper plasmid only with pTV00 derivatives) pJIC SA_Rep; kindly provided by the Sainsbury Laboratory, University of Cambridge, United Kingdom] were used.

The TRV PpK20 based vectors pBINTRA6, pTV00 and pTVPD [pTV00 carrying in its polylinker a 206 bp *N. benthamiana phytoene desaturase gene* (*pds*) fragment in antisense orientation] were kindly provided by the laboratory of Sir David Baulcombe.

Antibiotics were obtained from Duchefa (www.duchefa-biochemie.com). Kanamycin (50 mg/L) was used for plasmid selection in *E. coli* TOP10. Kanamycin (50 mg/L) and rifampicin (100 mg/L) were the selective antibiotics for the *A. tumefaciens* GV3101 strains. SuperScript™ III Reverse Transcriptase (Invitrogen, www.thermofisher.com) was the enzyme for cDNA synthesis. DNaseI (ThermoFisher Scientific, www.thermofisher.com) was used for removing DNA contaminations from RNA preparations. Phusion High-Fidelity DNA Polymerase (ThermoFisher Scientific, www.thermofisher.com) was the enzyme for polymerase chain reactions (PCR). Restriction enzymes were obtained from NEB (international.neb.com); T4-DNA ligase came from Invitrogen (www.thermofisher.com). All enzymes were used according to the instructions of the manufacturers. All oligonucleotides were synthesized by Sigma-Aldrich (sigmaaldrich.com).

### Amplification, isolation and sequencing of the TRV California genome

We obtained the virus in dried spinach leaf samples. The host plant species, e.g. spinach, pepper, *N. benthamiana* and *N. attenuata* were inoculated with TRV California by rubbing Celite (diatomaceous earth; Sigma-Aldrich, www.sigmaaldrich.com) mixed with powdered infected spinach leaf samples or soaked with the extracted plant juice of previously infected plants, with fingers into the leaves of the healthy seedlings. The total RNAs of the plants showing typical symptoms of TRV infection were isolated from leaf samples using Trizol reagent (Invitrogen, www.thermofisher.com) according to the instructions of the manufacturer, followed by DNaseI treatment, cDNA synthesis and PCR amplification. The primers for cDNA synthesis and PCR were designed from conserved sequences of full-length TRV-RNA1 and TRV-RNA2 genomes available in GenBank (Supplemental Table S1). First, the identity of the virus as TRV was confirmed by sequencing of a 341 bp and a 611 bp PCR fragment amplified from RNA1 cDNA. For this, cDNA was synthesized with an equimolar mixture of primers TRV40 and TRV45, followed by PCR with primer pairs TRV44/TRV45, and TRV25/TRV26, respectively, and the synthesized cDNA as template. In the next step, the full-length TRV California genomes were characterized. For this, the above described RNA1 cDNA and RNA2 cDNA synthesized with primer TRV52 were used. RNA1 and RNA2 genomes were then PCR-amplified with primer pairs TRV43/TRV40 and TRV51/TRV52, respectively. The full-length TRV RNA2 cDNA was cloned in the pCR Blunt II-TOPO vector (Invitrogen, www.thermofisher.com), yielding pCR-RNA2. Cloning of the full length TRV RNA1 was not possible since one TRV RNA1 encoded protein (probably the RdR) is toxic to *E. coli* [1]. Therefore, the complete TRV RNA1 was amplified from the RNA1 cDNA as three fragments with overlapping sequences using primer pairs TRV43/TRV24, TRV46/TRV47 and TRV44/TRV40. These fragments were sub-cloned in the pJET1.2 vector (ThermoFisher, www.thermofisher.com). All cloned TRV cDNA sequences were Sanger sequenced on an Applied Biosystems 3130xl Genetic Analyzer. The primers used for cDNA synthesis, PCR and sequencing are listed in Supplemental Table S2.

### Construction of TRV California VIGS vectors

To construct the new VIGS vectors, we used the backbone of plasmid pSOL9DEF1 (GenBank KF939593) and the cloned TRV California cDNA as PCR template. For the vector with the RNA1 cDNA, a series of consecutive cloning steps was performed: The 3′ terminal 1100 nt of RNA1 containing the 16 kDa protein ORF were amplified with primer pair TRV57/TRV58 and cloned as *Ase*I-*Bam*HI fragment in pSOL9DEF1 (pTRV11). The adjacent 589 nt of RNA1 were amplified with primer pair TRV59/TRV60 and cloned as *Pst*I-*Xho*I fragment (pTRV12). The next adjacent 2959 nt of RNA1 were amplified with primer pair TRV61/TRV60 and delivered as *Pst*I-*Sac*I fragment (pTRV13). The intron sequence of the *A. thaliana* NIA1 gene for nitrate reductase (Z19050) present on pBINTRA6 was amplified with primer pair TRV62/TRV63, flanking RNA1 was added to the PCR fragment by PCR with primer pairs TRV64/TRV63 and then TRV65/TRV63 (template preceding PCR-fragments). The final intron fragment was cloned as *Bst*EII-*Bgl*II fragment in RNA1 on pTRV13 to interrupt the ORF of the RdR and enable plasmid replication in *E. coli* (pTRV14). Further adjacent 242 nt of RNA1 were amplified with primer pair TRV66/TRV67 and cloned as *Apa*I-*Xba*I-fragment in pTRV14 (pTRV15). The RNA1 sequence was completed by delivering the residual 5′ 1875 nt of RNA1 as PCR fragment synthesized with primer pair TRV68/TRV69 and digested with *Pst*I and *Apa*I to pTRV15 (pTRV16). The 35S terminator was PCR amplified from pSOL9DEF1 with primer pair TRV70/TRV71 and added as 210 bp *Sna*BI-*Spe*I fragment to pTRV16 (TRV17). Finally, the 35S promoter was PCR amplified from pSOL9DEF1 with primer pair TRV72/TRV73 and cloned as 601 bp *Pme*I-*Pst*I fragment in pTRV17. The resulting VIGS vector was named pTRV-RNA1 and carries between CaMV35S promoter and terminator the full-length coding sequence of the TRV California RNA1 in sense orientation with an intron in the RdR ORF (14.4 kb, Fig. 3A).

To construct the vector with TRV California RNA2 cDNA, in the first step the complete RNA1 on pTRV-RNA1 was replaced with a polylinker followed by the 3′ 568 nt of RNA2 in sense orientation. For this, the PCR fragment obtained with primer pair TRV74/TRV75 was cloned as *Pst*I-*Xho*I fragment in pTRV-RNA1, yielding pTRV21. In the second step, the PCR fragment synthesized with primer pair TRV76/TRV77, comprising the 5′ 1161 nt of RNA2 with the ORF of the coat protein was delivered as *Pst*I-*Sal*I fragment to pTRV21. The obtained VIGS vector pTRV-RNA2 (8.9 kb, Fig. 3B) carried under the control of CaMV35S promoter and terminator the ORF of the TRV coat protein, directly downstream followed by a polylinker to facilitate cloning of target gene fragments. This polylinker was used to clone a 206 bp PCR fragment of the *phytoene desaturase (PDS*) gene from *N. benthamiana* and a 258 bp PCR fragment of the *allene oxide cyclase (AOC*) gene from *N. attenuata* as *Kpn*I-*Sac*I fragments on pTRV-RNA2, yielding pTRV-RNA2:PDS (9.1 kb) and pTRV:RNA2:AOC (9.2 kb). PCR was performed with primer pair PDS43/PDS44 on template pTVPD and with primer pair AOC7/AOC8 on cDNA from *N. attenuata* as template. The primers used for plasmid construction and sequencing are listed in Supplemental Table S2.

### VIGS inoculation of *Nicotiana attenuata*

*Agrobacterium tumefaciens* GV3101 was transformed with plasmids pBINTRA6, pTV00, pTVPD, pTRV-RNA1, pTRV-RNA2, pTRV-RNA2:PDS and pTRV-RNA2:AOC. Leaves of 23–25 days old, healthy *N. attenuata* seedlings were co-inoculated with an *A. tumefaciens* GV3101 carrying a plasmid with TRV genome 1 (pBINTRA6 or pTRV-RNA1) and *A. tumefaciens* GV3101 carrying a plasmid with TRV genome 2 (pTV00, pTVPD, pTRV-RNA2, pTRV-RNA2:PDS or pTRV-RNA2:AOC) using a 1 mL syringe without needle following the protocol described by Galis et al. (2013) [3]. For plant-juice inoculation, the juice was first extracted from the leaves of pre-established PDS silenced plants (*N. attenuata* previously co-inoculated with *A. tumefaciens* GV3101 carrying pBINTRA6/pTVPD or pTRV-RNA1/pTRV-RNA2:PDS). The extracted leave juice was then mixed with a tiny amount of Celite and young leaves of 23–25 days old, healthy plants were inoculated by rubbing. After inoculation, the plants were covered with an upside-down black tray and left in the dark for 2 days at 22 °C to allow for *A. tumefaciens* proliferation and transformation. For the comparison of silencing efficiency of the two VIGS-vector systems (pBINTRA6/pTV00 and pTRV-RNA1/pTRV-RNA2) at higher temperatures, inoculated plants were then transferred to growth chambers maintained at temperatures of 26 °C, 28 °C, 30 °C and 32 °C. At thirteen dpi (day post inoculation) apical leaf samples of inoculated plants were harvested for the determination of silencing efficiency of the target genes *PDS* and *AOC*. All samples were collected in 2 mL Eppendorf tubes, flash frozen in liquid nitrogen and stored at − 80 °C until analysis.

### Treatment of *N. attenuata* with oral secretion of *Manduca sexta*

*Manduca sexta* oral secretion (OS) was collected on ice from larvae reared on *N. attenuata* plants until third to fifth instar as previously described [54]. For the induction of *AOC* transcripts in plants inoculated with the pTRV-RNA2:AOC and pTRV-RNA2 empty vector (EV) control constructs, at 13 dpi, one leaf per plant was wounded with a pattern wheel, and 20 μL of 1:5 diluted OS of *Manduca sexta* were added to the puncture wounds. The apical leaves samples were harvested for quantification of *AOC* transcripts 5 h after OS treatment.

### Quantitative PCR (qPCR) analysis

Total RNA was isolated from leaf samples using Trizol reagent (Invitrogen, www.thermofisher.com) according to the instructions of the manufacturer, followed by DNaseI treatment. For cDNA preparation, 2 μg of DNase-treated total RNA were used for first strand cDNA synthesis with an oligo (dT)_18_ primer. For quantitative PCR analysis, four independent biological and three technical replicate samples were used for each treatment. Quantitative PCR analyses were performed on a Stratagene MX3005P (Agilent Technologies, www.agilent.com) using a qPCR Core Kit for SYBR Green I according to the instructions of the manufacturer (Eurogentec, www.eurogentec.com). The primers used for qPCR are listed in Supplemental Table S2. The elongation factor 1-alpha (EF1) gene (GenBank XM_019399807) was used for normalization.

### Sequence deposition in GenBank

Sequence data of full-length genomes of TRV California RNA1 (MH614641) and RNA2 (MH614642) and the two VIGS vectors pTRV-RNA1 (MH625695) and pTRV-RNA2 (MH625696) are have been submitted to GenBank.

## Supplementary Information

**Additional file 1: Supplementary Table S1.** GenBank accessions of full-length RNA1 and RNA2 genomes of different TRV isolates. **Supplementary Table S2**. List of primers used. **Supplementary Table S3**. Effect of different growth temperatures on *PDS* gene silencing in *N. attenuata* induced by TRV California VIGS vectors (pTRV-RNA1/pTRV-RNA2:PDS). **Supplementary Table S4.** Monthly temperatures in Santa Barbara, CA in 2009. **Supplementary Table S5.** Monthly average temperatures in Scotland 1971–2000. **Supplementary file 2:** Supplementary Figures. **Supplementary figure S1.** Disease symptoms in host plant species mechanically infected with TRV California. **Supplementary figure S2.** Detection of the TRV infection in host plants. **Supplementary Figure S3.** *N. attenuata* plants inoculated with the TRV California vector system, grown at different temperatures. **Supplementary figure S4.** Systemic silencing of the *PDS* gene in *N. attenuata* induced with the TRV California and PpK20 vectors after growth at 28°C and 30°C (sap inoculated). **Supplementary figure S5.** Swapping of RNA1 and RNA2 vectors of California and PpK20 isolates. **Supplementary figure S6.** ClustalW analysis of the RNA dependent RNA polymerase (RdR) proteins from the TRV California and TRV PpK20 isolates**. Supplementary figure S7.** ClustalW analysis of the 16 kDa Suppressor proteins (A), Movement proteins (B) and Coat proteins (C) from the TRV California and TRV PpK20 isolates. **Supplementary file 3:** Original gel images of Fig. 1D, E, S2A and S2B with legends. **Supplementary file 4:** Original gel image file (JPEG format) Fig. 1D. **Supplementary file 5:** Original gel image file (JPEG format) Fig. 1E. **Supplementary file 6:** Original gel image file (JPEG format) Fig. S2A. **Supplementary file 7:** Original gel image file (JPEG format) Fig. S2B.

## Data Availability

The authors declare that TRV California infected spinach leaf samples and the new VIGS vectors, TRV-RNA1 and TRV-RNA2, are available upon request.

## Abbreviations

AOC: Allene oxide cyclase
CaMV: Cauliflower Mosaic Virus
dpi: Days post inoculation
EF1: Elongation factor 1-alpha
EV: Empty vector
NCR: Non-coding regions
nt: Nucleotides
ORF: Open reading frame
OS: Oral secretion
PDS: Phytoene desaturase
RdR: RNA-dependent RNA polymerase
TRV: Tobacco Rattle Virus
VIGS: Virus-induced gene silencing
WT: Wild type
