# Supplementary material for: California TRV-based VIGS vectors mediate gene silencing at elevated temperatures but with greater growth stunting
Source: BMC Plant Biol. 2021 Nov 22;21:553. doi: 10.1186/s12870-021-03324-8 (PMC8607596; doi:10.1186/s12870-021-03324-8)
Supplement: Supplementary file 1 — Additional file 1: Supplementary Table S1. GenBank accessions of full-length RNA1 and RNA2 genomes of different TRV isolates. Supplementary Table S2. List of primers used. Supplementary Table S3. Effect of different growth temperatures on PDS gene silencing in N. attenuata induced by TRV California VIGS vectors (pTRV-RNA1/pTRV-RNA2:PDS). Supplementary Table S4. Monthly temperatures in Santa Barbara, CA in 2009. Supplementary Table S5. Monthly average temperatures in Scotland 1971–2000. Supplementary file 2: Supplementary Figures. Supplementary figure S1. Disease symptoms in host plant species mechanically infected with TRV California. Supplementary figure S2. Detection of the TRV infection in host plants. Supplementary Figure S3. N. attenuata plants inoculated with the TRV California vector system, grown at different temperatures. Supplementary figure S4. Systemic silencing of the PDS gene in N. attenuata induced with the TRV California and PpK20 vectors after growth at 28°C and 30°C (sap inoculated). Supplementary figure S5. Swapping of RNA1 and RNA2 vectors of California and PpK20 isolates. Supplementary figure S6. ClustalW analysis of the RNA dependent RNA polymerase (RdR) proteins from the TRV California and TRV PpK20 isolates. Supplementary figure S7. ClustalW analysis of the 16 kDa Suppressor proteins (A), Movement proteins (B) and Coat proteins (C) from the TRV California and TRV PpK20 isolates. Supplementary file 3: Original gel images of Fig. 1D, E, S2A and S2B with legends. Supplementary file 4: Original gel image file (JPEG format) Fig. 1D. Supplementary file 5: Original gel image file (JPEG format) Fig. 1E. Supplementary file 6: Original gel image file (JPEG format) Fig. S2A. Supplementary file 7: Original gel image file (JPEG format) Fig. S2B. [file 12870_2021_3324_MOESM1_ESM.zip › Original gel images of Figures 1D, 1E, S2A and S2B.pptx]

## Slide 1
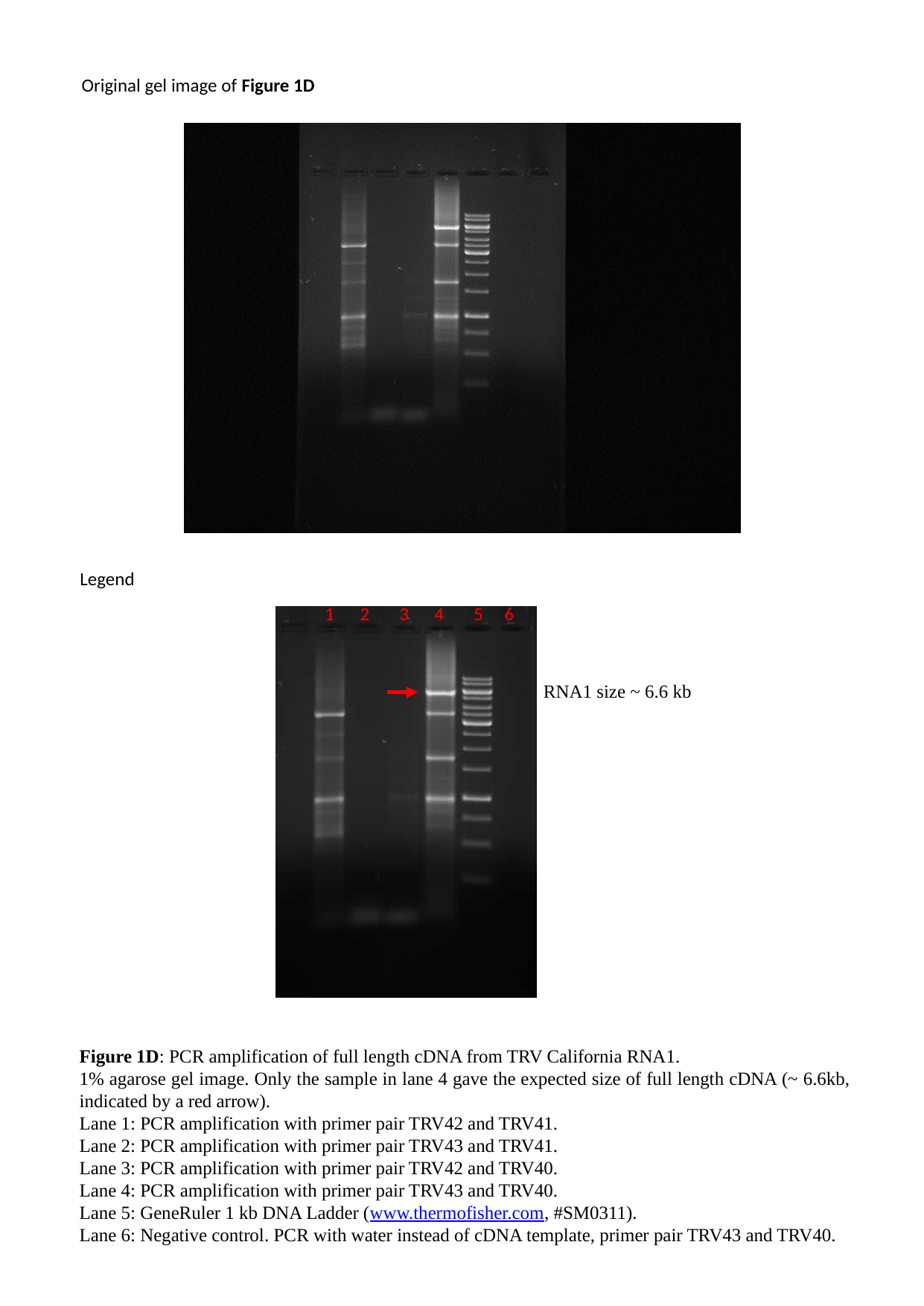

Original gel image of Figure 1D
Legend
 1 2 3 4 5 6
RNA1 size ~ 6.6 kb
Figure 1D: PCR amplification of full length cDNA from TRV California RNA1.
1% agarose gel image. Only the sample in lane 4 gave the expected size of full length cDNA (~ 6.6kb, indicated by a red arrow).
Lane 1: PCR amplification with primer pair TRV42 and TRV41.
Lane 2: PCR amplification with primer pair TRV43 and TRV41.
Lane 3: PCR amplification with primer pair TRV42 and TRV40.
Lane 4: PCR amplification with primer pair TRV43 and TRV40.
Lane 5: GeneRuler 1 kb DNA Ladder (www.thermofisher.com, #SM0311).
Lane 6: Negative control. PCR with water instead of cDNA template, primer pair TRV43 and TRV40.

## Slide 2
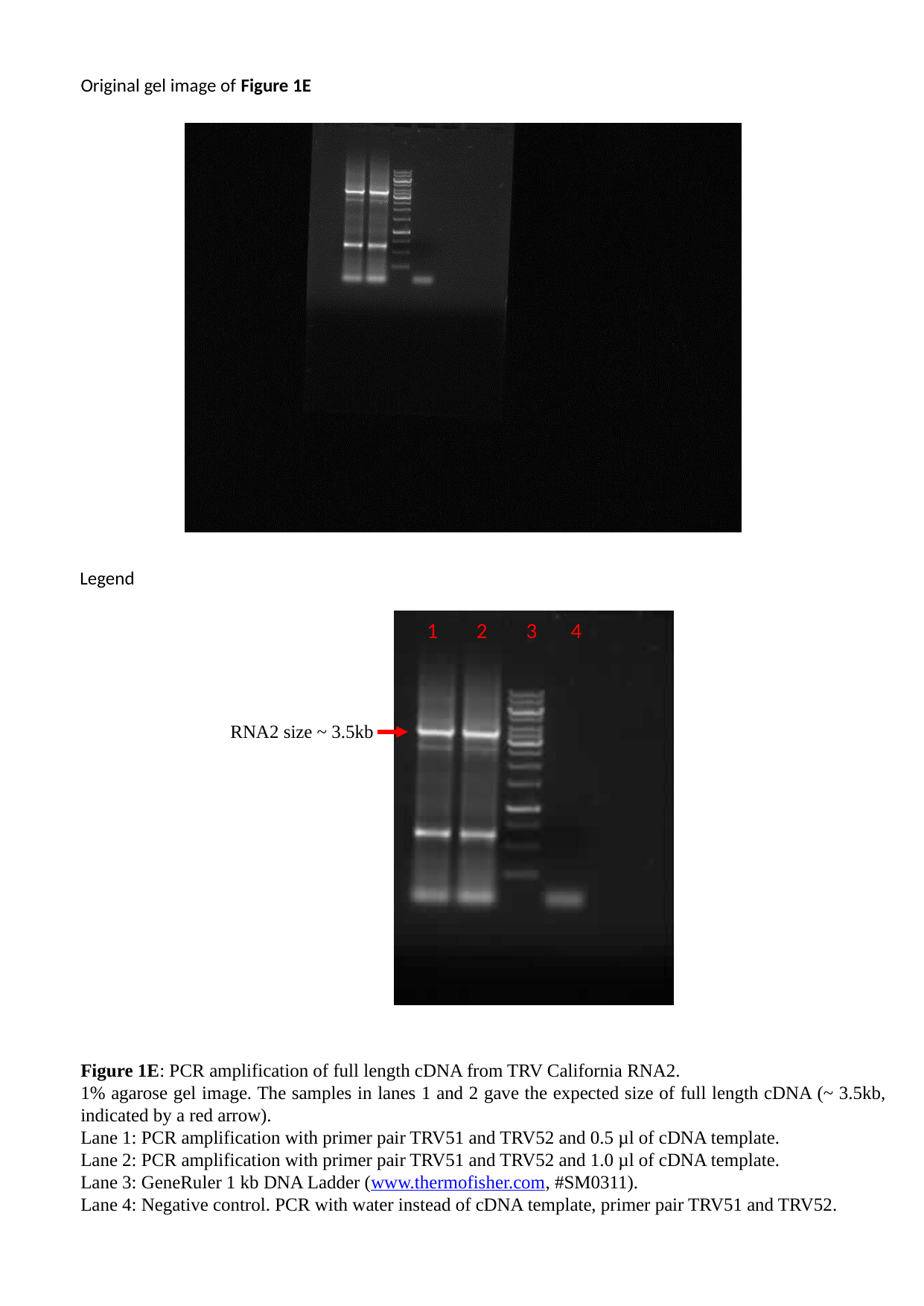

Original gel image of Figure 1E
Legend
 1 2 3 4
RNA2 size ~ 3.5kb
Figure 1E: PCR amplification of full length cDNA from TRV California RNA2.
1% agarose gel image. The samples in lanes 1 and 2 gave the expected size of full length cDNA (~ 3.5kb, indicated by a red arrow).
Lane 1: PCR amplification with primer pair TRV51 and TRV52 and 0.5 µl of cDNA template.
Lane 2: PCR amplification with primer pair TRV51 and TRV52 and 1.0 µl of cDNA template.
Lane 3: GeneRuler 1 kb DNA Ladder (www.thermofisher.com, #SM0311).
Lane 4: Negative control. PCR with water instead of cDNA template, primer pair TRV51 and TRV52.

## Slide 3
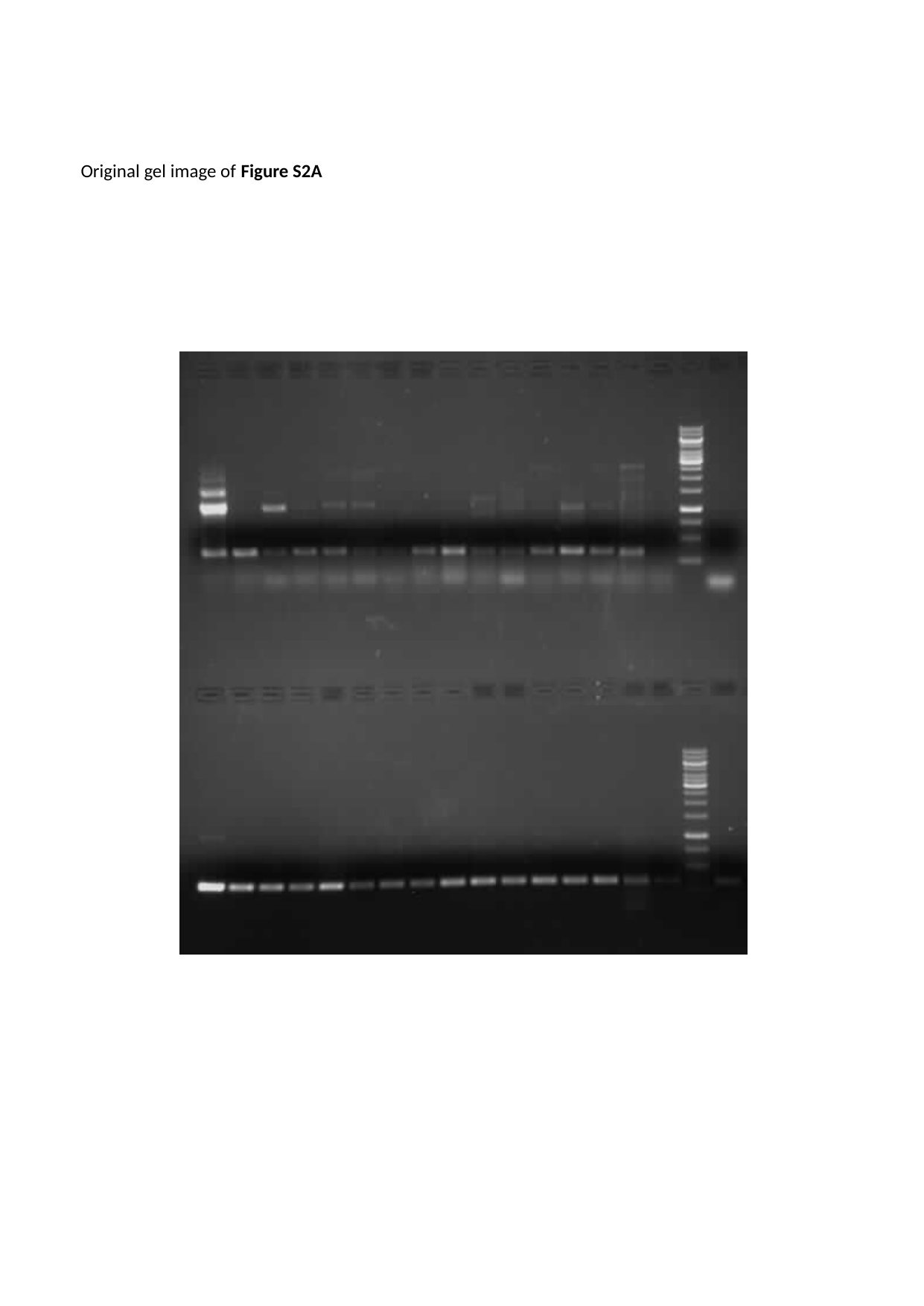

Original gel image of Figure S2A

## Slide 4
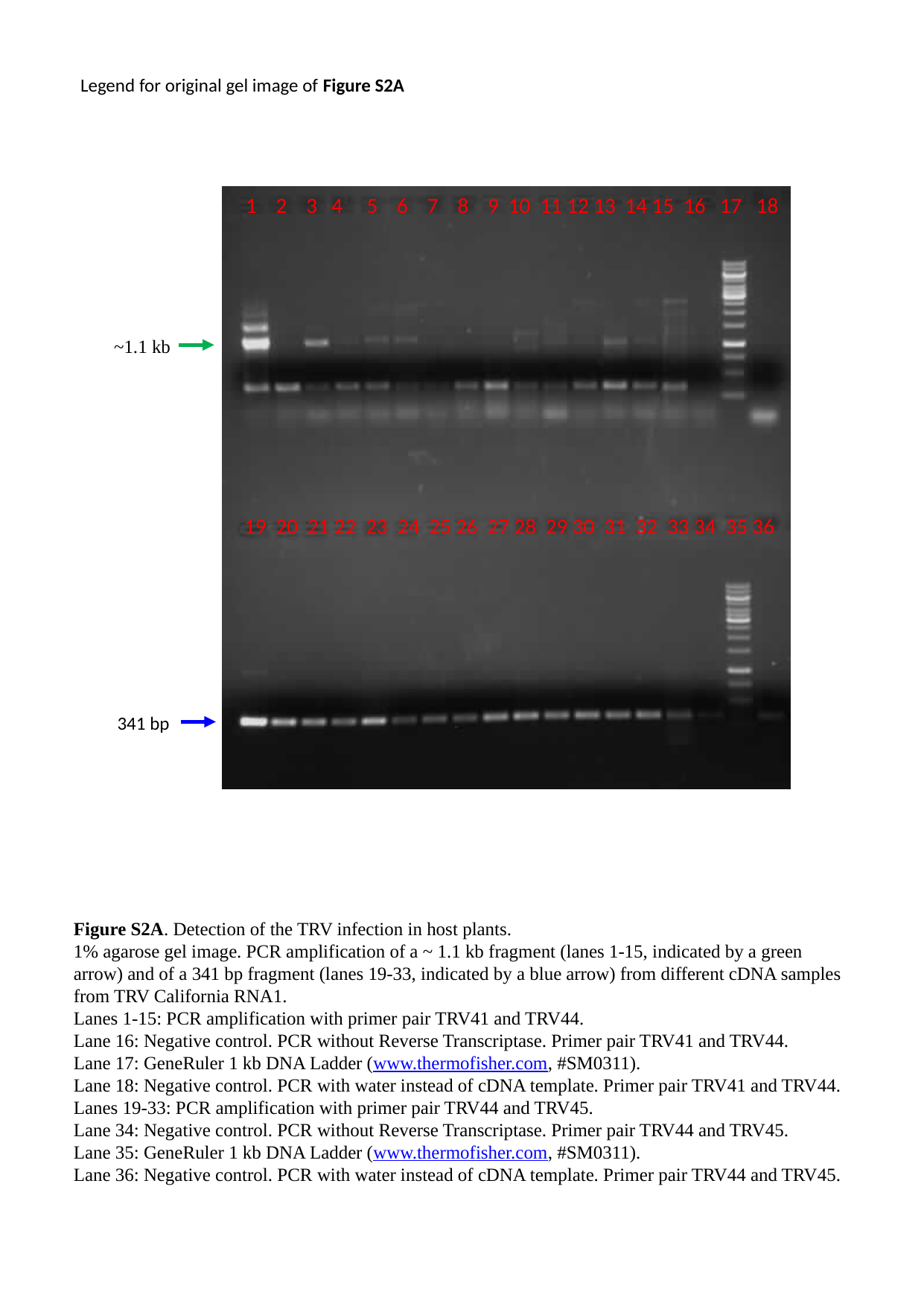

Legend for original gel image of Figure S2A
1 2 3 4 5 6 7 8 9 10 11 12 13 14 15 16 17 18
~1.1 kb
19 20 21 22 23 24 25 26 27 28 29 30 31 32 33 34 35 36
341 bp
Figure S2A. Detection of the TRV infection in host plants.
1% agarose gel image. PCR amplification of a ~ 1.1 kb fragment (lanes 1-15, indicated by a green arrow) and of a 341 bp fragment (lanes 19-33, indicated by a blue arrow) from different cDNA samples from TRV California RNA1.
Lanes 1-15: PCR amplification with primer pair TRV41 and TRV44.
Lane 16: Negative control. PCR without Reverse Transcriptase. Primer pair TRV41 and TRV44.
Lane 17: GeneRuler 1 kb DNA Ladder (www.thermofisher.com, #SM0311).
Lane 18: Negative control. PCR with water instead of cDNA template. Primer pair TRV41 and TRV44.
Lanes 19-33: PCR amplification with primer pair TRV44 and TRV45.
Lane 34: Negative control. PCR without Reverse Transcriptase. Primer pair TRV44 and TRV45.
Lane 35: GeneRuler 1 kb DNA Ladder (www.thermofisher.com, #SM0311).
Lane 36: Negative control. PCR with water instead of cDNA template. Primer pair TRV44 and TRV45.

## Slide 5
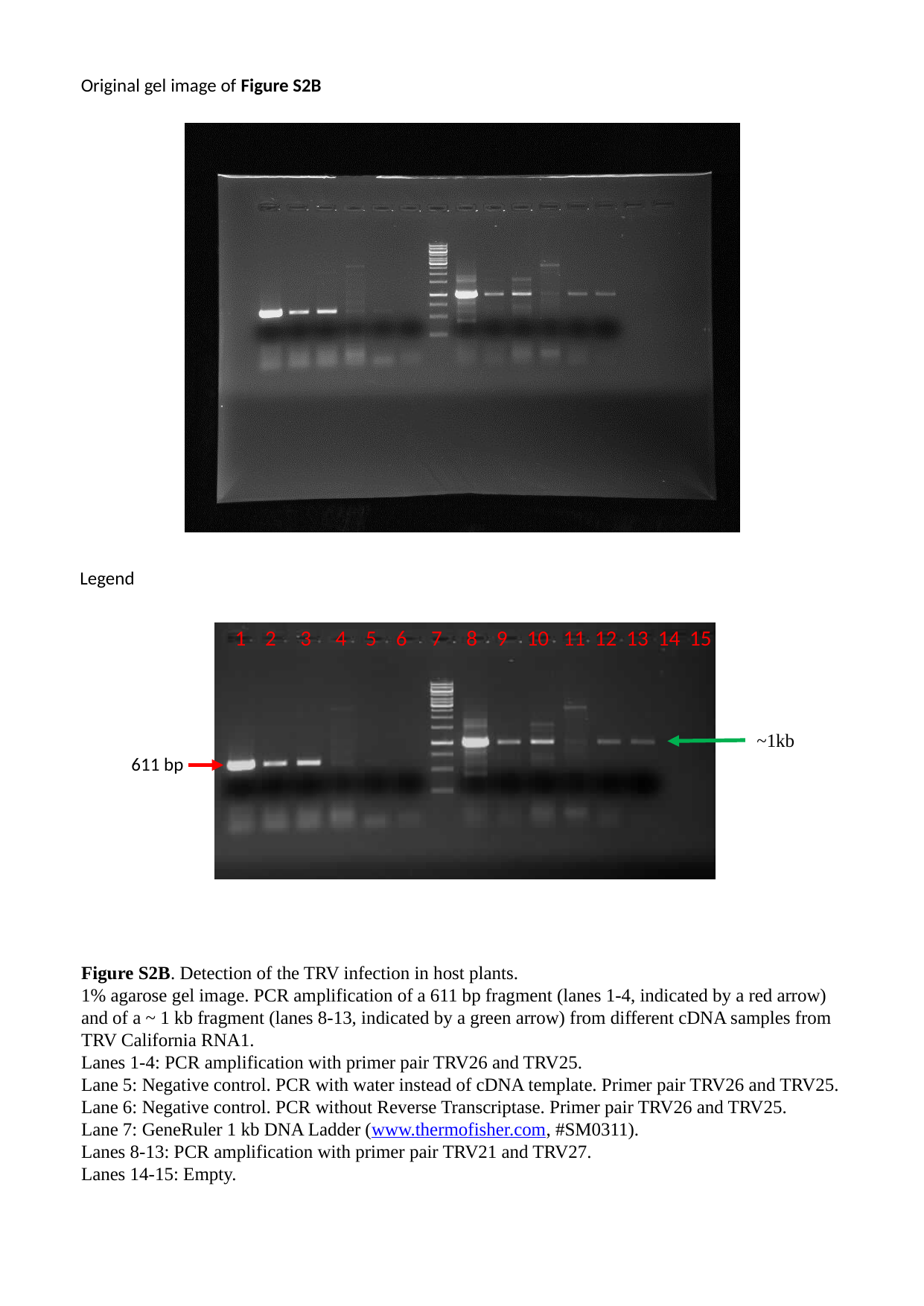

Original gel image of Figure S2B
Legend
1 2 3 4 5 6 7 8 9 10 11 12 13 14 15
~1kb
611 bp
Figure S2B. Detection of the TRV infection in host plants.
1% agarose gel image. PCR amplification of a 611 bp fragment (lanes 1-4, indicated by a red arrow) and of a ~ 1 kb fragment (lanes 8-13, indicated by a green arrow) from different cDNA samples from TRV California RNA1.
Lanes 1-4: PCR amplification with primer pair TRV26 and TRV25.
Lane 5: Negative control. PCR with water instead of cDNA template. Primer pair TRV26 and TRV25.
Lane 6: Negative control. PCR without Reverse Transcriptase. Primer pair TRV26 and TRV25.
Lane 7: GeneRuler 1 kb DNA Ladder (www.thermofisher.com, #SM0311).
Lanes 8-13: PCR amplification with primer pair TRV21 and TRV27.
Lanes 14-15: Empty.
