# Supplementary material for: California TRV-based VIGS vectors mediate gene silencing at elevated temperatures but with greater growth stunting
Source: BMC Plant Biol. 2021 Nov 22;21:553. doi: 10.1186/s12870-021-03324-8 (PMC8607596; doi:10.1186/s12870-021-03324-8)
Supplement: Supplementary file 1 — Additional file 1: Supplementary Table S1. GenBank accessions of full-length RNA1 and RNA2 genomes of different TRV isolates. Supplementary Table S2. List of primers used. Supplementary Table S3. Effect of different growth temperatures on PDS gene silencing in N. attenuata induced by TRV California VIGS vectors (pTRV-RNA1/pTRV-RNA2:PDS). Supplementary Table S4. Monthly temperatures in Santa Barbara, CA in 2009. Supplementary Table S5. Monthly average temperatures in Scotland 1971–2000. Supplementary file 2: Supplementary Figures. Supplementary figure S1. Disease symptoms in host plant species mechanically infected with TRV California. Supplementary figure S2. Detection of the TRV infection in host plants. Supplementary Figure S3. N. attenuata plants inoculated with the TRV California vector system, grown at different temperatures. Supplementary figure S4. Systemic silencing of the PDS gene in N. attenuata induced with the TRV California and PpK20 vectors after growth at 28°C and 30°C (sap inoculated). Supplementary figure S5. Swapping of RNA1 and RNA2 vectors of California and PpK20 isolates. Supplementary figure S6. ClustalW analysis of the RNA dependent RNA polymerase (RdR) proteins from the TRV California and TRV PpK20 isolates. Supplementary figure S7. ClustalW analysis of the 16 kDa Suppressor proteins (A), Movement proteins (B) and Coat proteins (C) from the TRV California and TRV PpK20 isolates. Supplementary file 3: Original gel images of Fig. 1D, E, S2A and S2B with legends. Supplementary file 4: Original gel image file (JPEG format) Fig. 1D. Supplementary file 5: Original gel image file (JPEG format) Fig. 1E. Supplementary file 6: Original gel image file (JPEG format) Fig. S2A. Supplementary file 7: Original gel image file (JPEG format) Fig. S2B. [file 12870_2021_3324_MOESM1_ESM.zip › Rahman et al Supplementary Figures.pptx]

## Slide 1
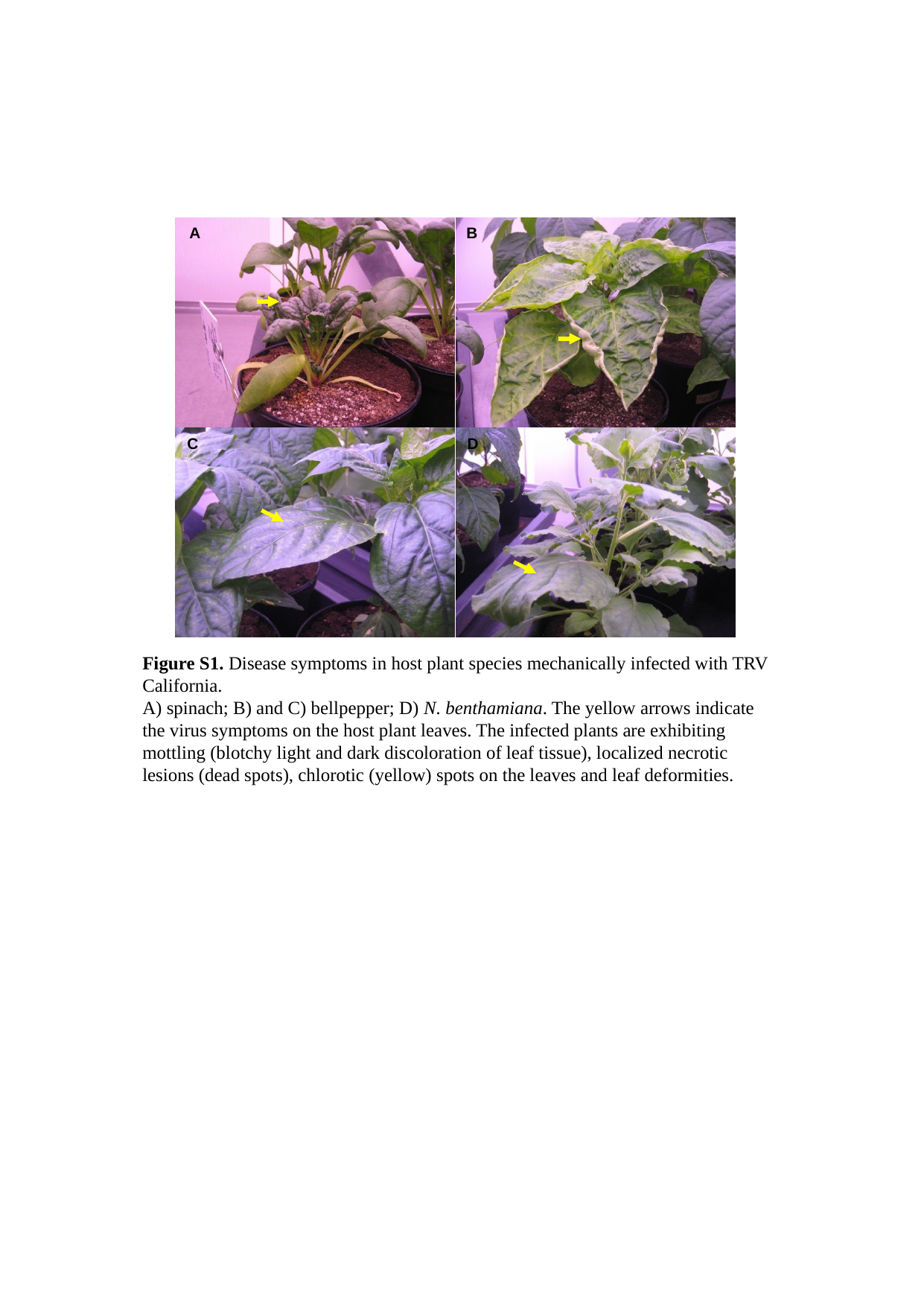

A
B
C
D
Figure S1. Disease symptoms in host plant species mechanically infected with TRV California.
A) spinach; B) and C) bellpepper; D) N. benthamiana. The yellow arrows indicate the virus symptoms on the host plant leaves. The infected plants are exhibiting mottling (blotchy light and dark discoloration of leaf tissue), localized necrotic lesions (dead spots), chlorotic (yellow) spots on the leaves and leaf deformities.

## Slide 2
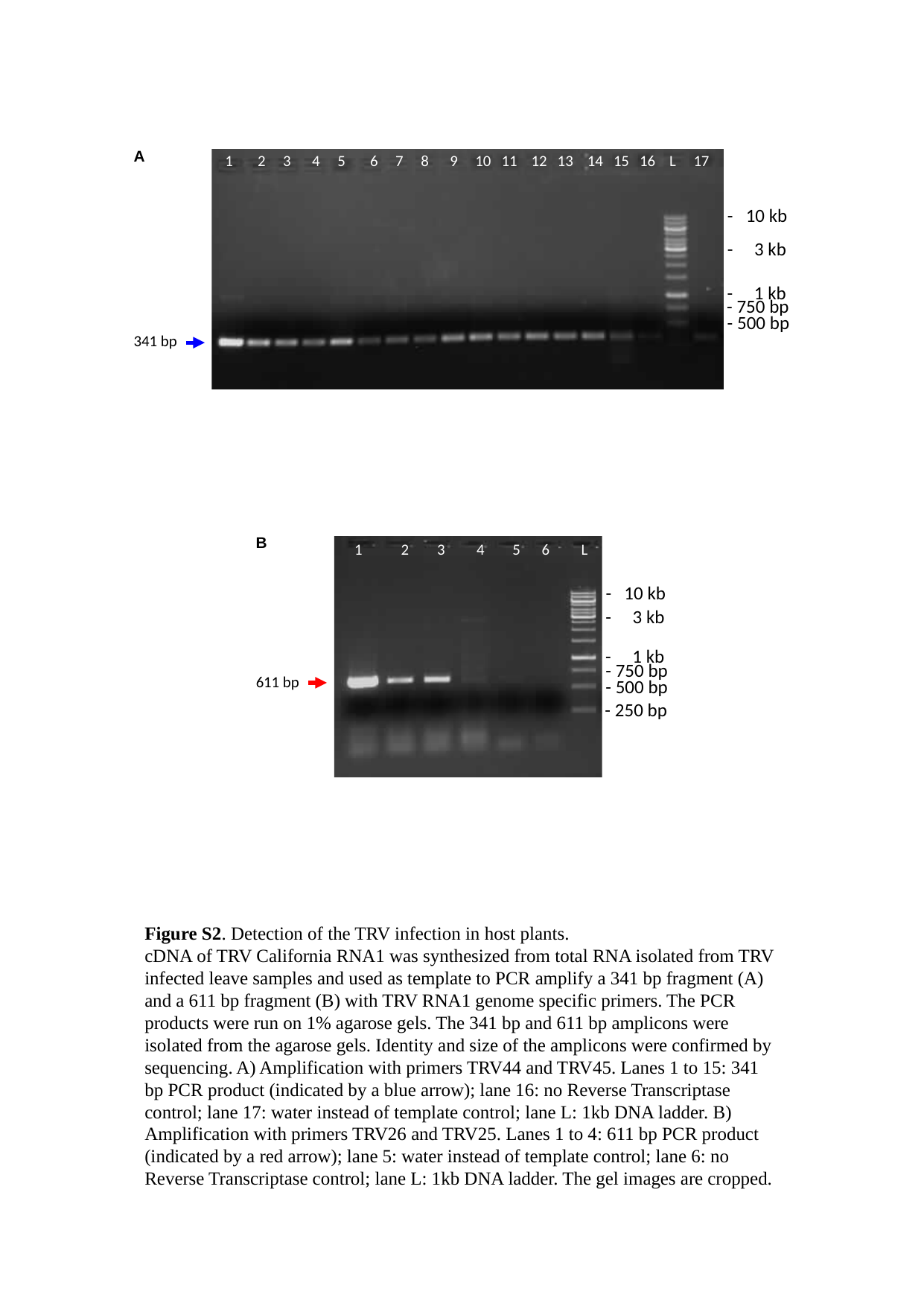

A
1 2 3 4 5 6 7 8 9 10 11 12 13 14 15 16 L 17
- 10 kb
- 3 kb
- 1 kb
- 750 bp
- 500 bp
341 bp
B
1 2 3 4 5 6 L
- 10 kb
- 3 kb
- 1 kb
- 750 bp
611 bp
- 500 bp
- 250 bp
Figure S2. Detection of the TRV infection in host plants.
cDNA of TRV California RNA1 was synthesized from total RNA isolated from TRV infected leave samples and used as template to PCR amplify a 341 bp fragment (A) and a 611 bp fragment (B) with TRV RNA1 genome specific primers. The PCR products were run on 1% agarose gels. The 341 bp and 611 bp amplicons were isolated from the agarose gels. Identity and size of the amplicons were confirmed by sequencing. A) Amplification with primers TRV44 and TRV45. Lanes 1 to 15: 341 bp PCR product (indicated by a blue arrow); lane 16: no Reverse Transcriptase control; lane 17: water instead of template control; lane L: 1kb DNA ladder. B) Amplification with primers TRV26 and TRV25. Lanes 1 to 4: 611 bp PCR product (indicated by a red arrow); lane 5: water instead of template control; lane 6: no Reverse Transcriptase control; lane L: 1kb DNA ladder. The gel images are cropped.

## Slide 3
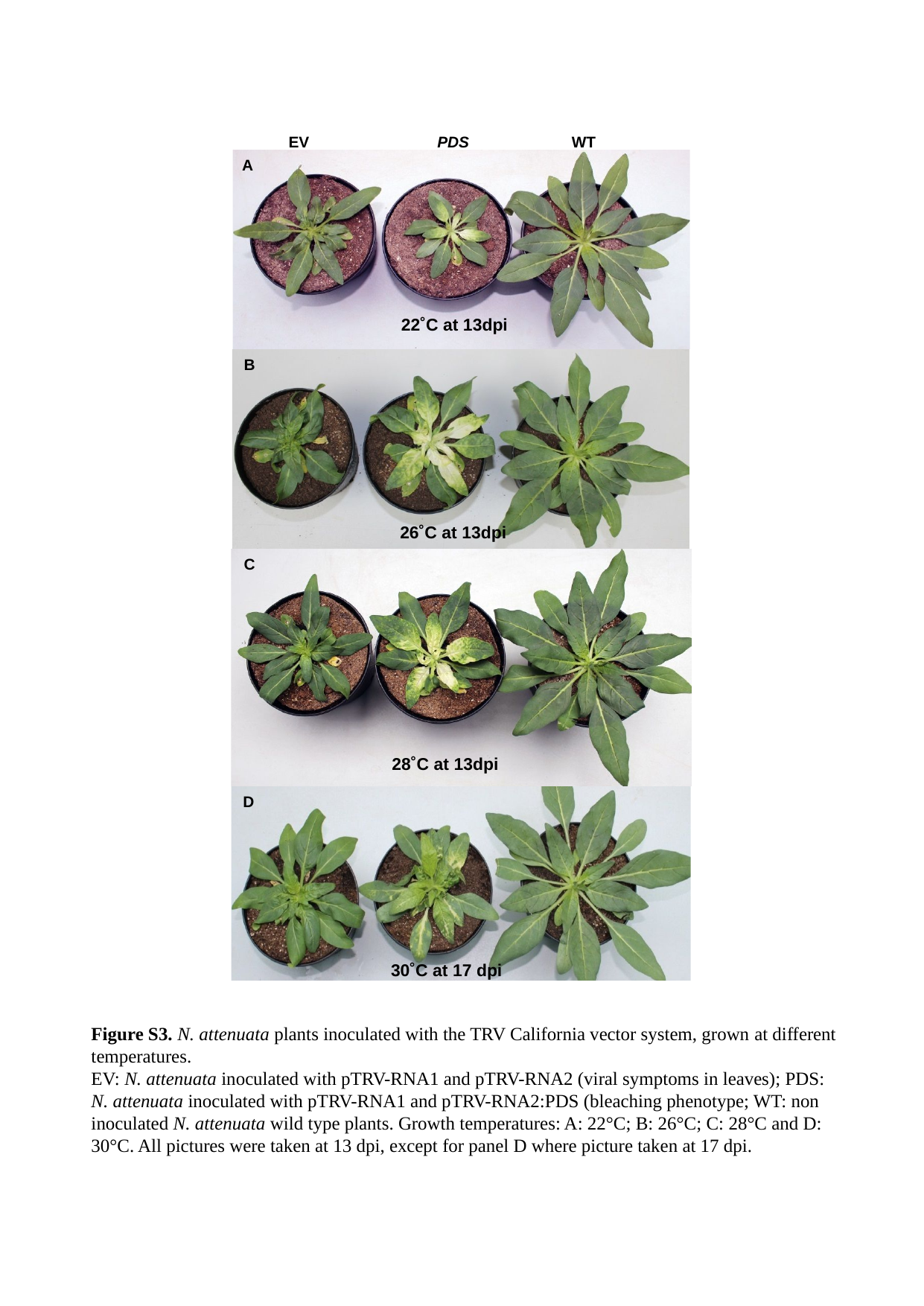

EV PDS WT
22˚C at 13dpi
26˚C at 13dpi
28˚C at 13dpi
30˚C at 17 dpi
A
B
C
D
Figure S3. N. attenuata plants inoculated with the TRV California vector system, grown at different temperatures.
EV: N. attenuata inoculated with pTRV-RNA1 and pTRV-RNA2 (viral symptoms in leaves); PDS: N. attenuata inoculated with pTRV-RNA1 and pTRV-RNA2:PDS (bleaching phenotype; WT: non inoculated N. attenuata wild type plants. Growth temperatures: A: 22°C; B: 26°C; C: 28°C and D: 30°C. All pictures were taken at 13 dpi, except for panel D where picture taken at 17 dpi.

## Slide 4
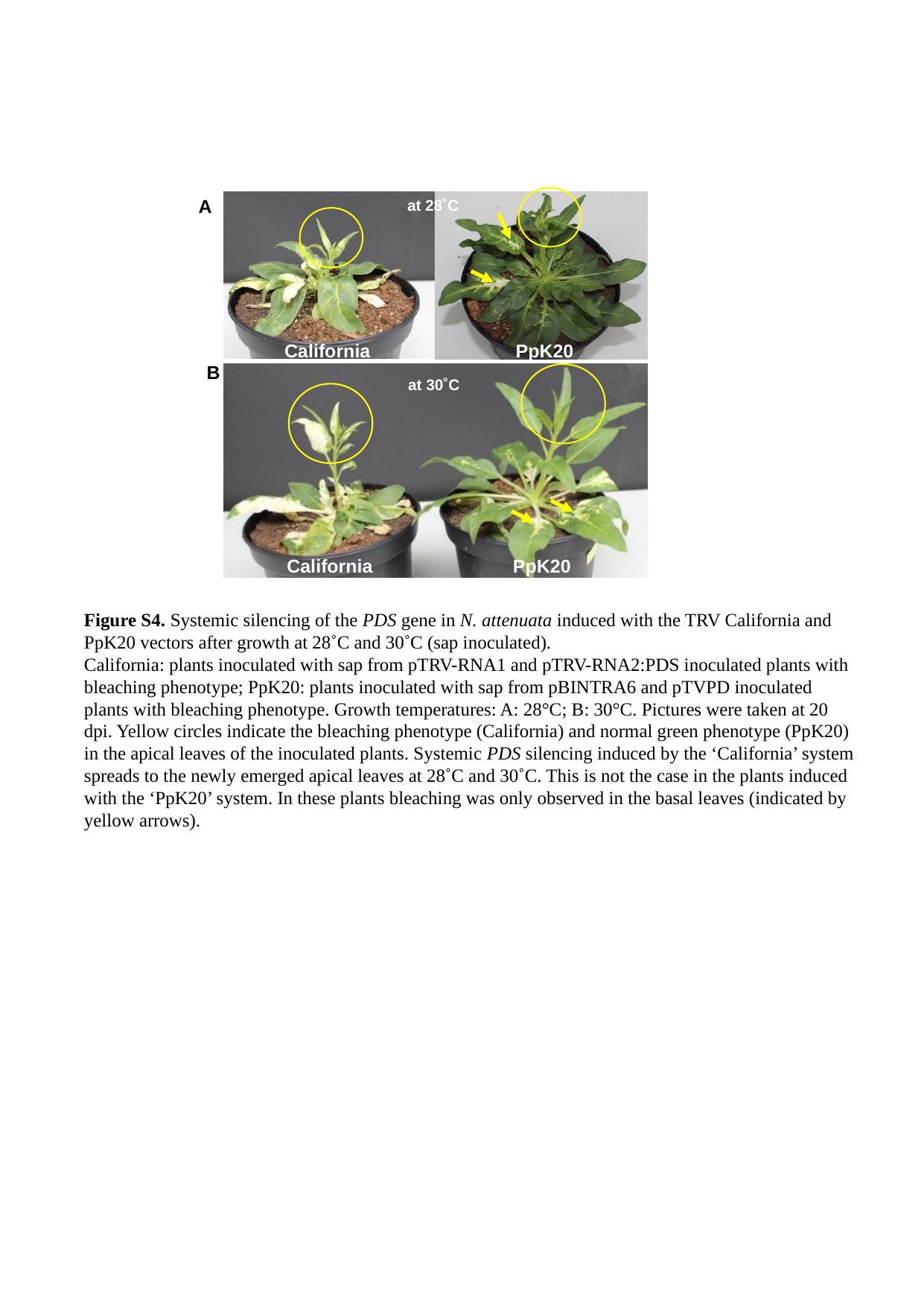

A
at 28˚C
 California PpK20
B
at 30˚C
at 30˚C
 California PpK20
Figure S4. Systemic silencing of the PDS gene in N. attenuata induced with the TRV California and PpK20 vectors after growth at 28˚C and 30˚C (sap inoculated).
California: plants inoculated with sap from pTRV-RNA1 and pTRV-RNA2:PDS inoculated plants with bleaching phenotype; PpK20: plants inoculated with sap from pBINTRA6 and pTVPD inoculated plants with bleaching phenotype. Growth temperatures: A: 28°C; B: 30°C. Pictures were taken at 20 dpi. Yellow circles indicate the bleaching phenotype (California) and normal green phenotype (PpK20) in the apical leaves of the inoculated plants. Systemic PDS silencing induced by the ‘California’ system spreads to the newly emerged apical leaves at 28˚C and 30˚C. This is not the case in the plants induced with the ‘PpK20’ system. In these plants bleaching was only observed in the basal leaves (indicated by yellow arrows).

## Slide 5
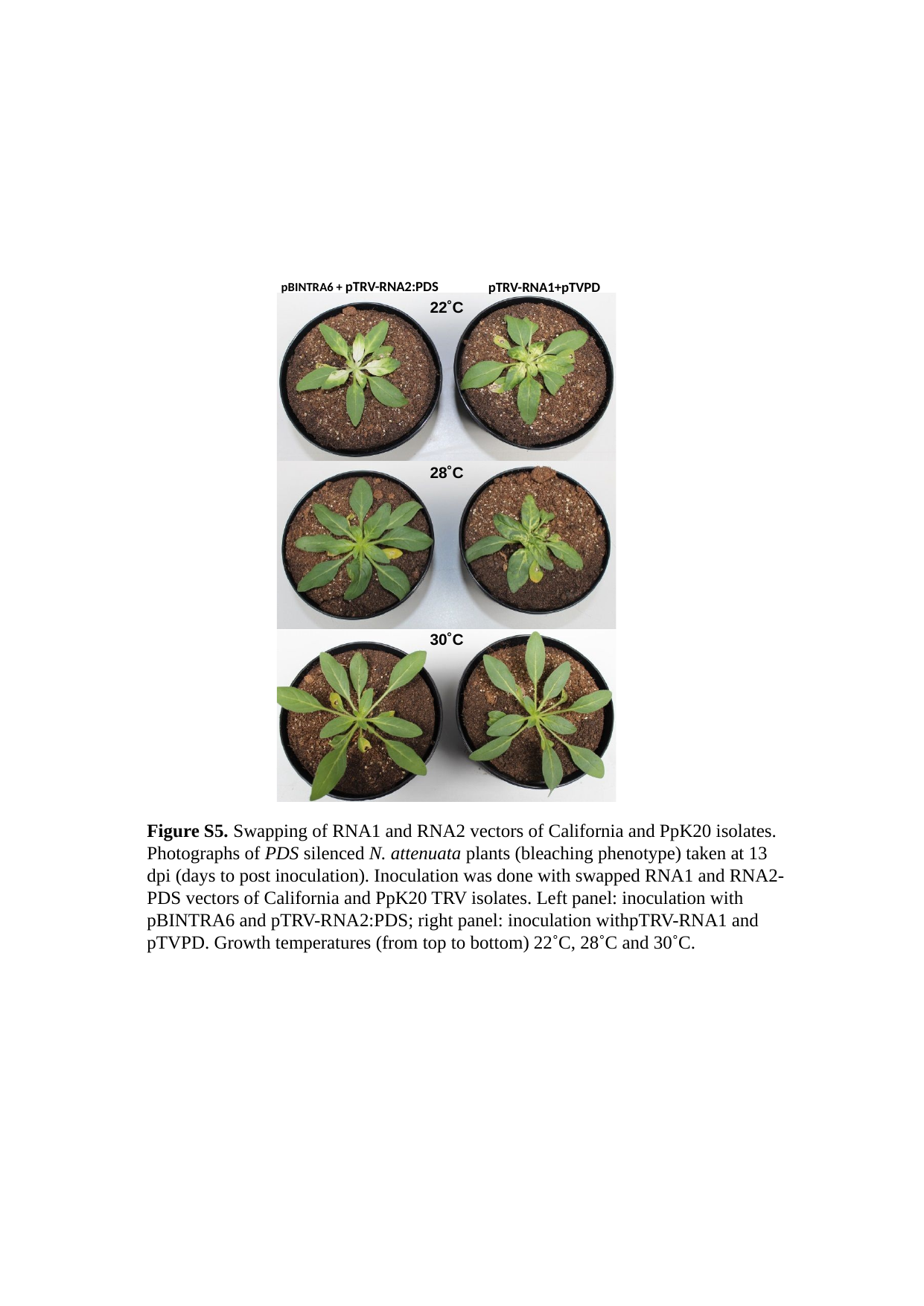

pBINTRA6 + pTRV-RNA2:PDS
pTRV-RNA1+pTVPD
22˚C
28˚C
30˚C
Figure S5. Swapping of RNA1 and RNA2 vectors of California and PpK20 isolates.
Photographs of PDS silenced N. attenuata plants (bleaching phenotype) taken at 13 dpi (days to post inoculation). Inoculation was done with swapped RNA1 and RNA2-PDS vectors of California and PpK20 TRV isolates. Left panel: inoculation with pBINTRA6 and pTRV-RNA2:PDS; right panel: inoculation withpTRV-RNA1 and pTVPD. Growth temperatures (from top to bottom) 22˚C, 28˚C and 30˚C.

## Slide 6
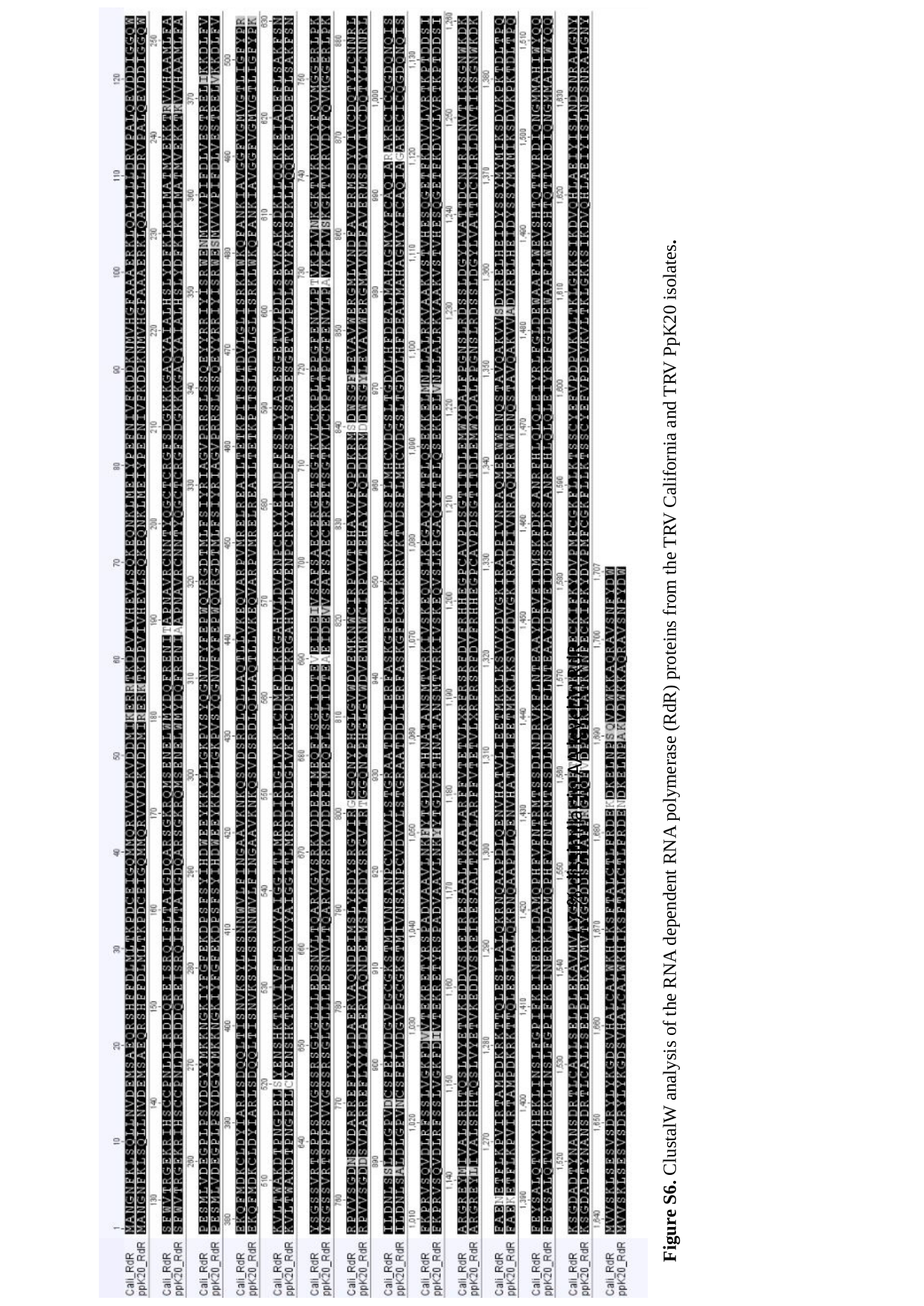

1.7 % dissimilar in AA level in RdR
Figure S6. ClustalW analysis of the RNA dependent RNA polymerase (RdR) proteins from the TRV California and TRV PpK20 isolates.

## Slide 7
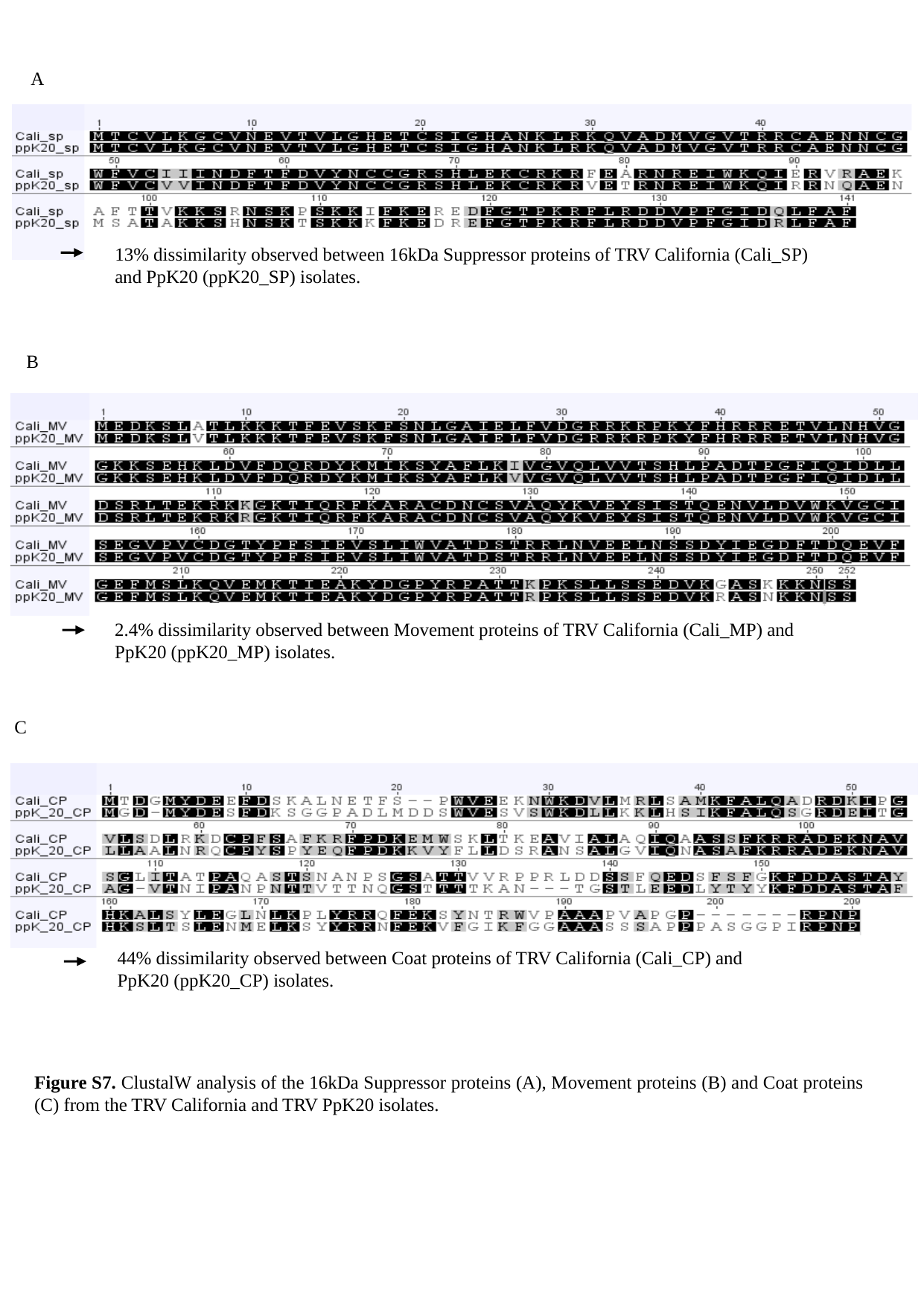

A
13% dissimilarity observed between 16kDa Suppressor proteins of TRV California (Cali_SP)
and PpK20 (ppK20_SP) isolates.
B
2.4% dissimilarity observed between Movement proteins of TRV California (Cali_MP) and
PpK20 (ppK20_MP) isolates.
C
44% dissimilarity observed between Coat proteins of TRV California (Cali_CP) and
PpK20 (ppK20_CP) isolates.
Figure S7. ClustalW analysis of the 16kDa Suppressor proteins (A), Movement proteins (B) and Coat proteins (C) from the TRV California and TRV PpK20 isolates.
