# Supplementary material for: California TRV-based VIGS vectors mediate gene silencing at elevated temperatures but with greater growth stunting
Source: BMC Plant Biol. 2021 Nov 22;21:553. doi: 10.1186/s12870-021-03324-8 (PMC8607596; doi:10.1186/s12870-021-03324-8)
Supplement: Supplementary file 1 — Additional file 1: Supplementary Table S1. GenBank accessions of full-length RNA1 and RNA2 genomes of different TRV isolates. Supplementary Table S2. List of primers used. Supplementary Table S3. Effect of different growth temperatures on PDS gene silencing in N. attenuata induced by TRV California VIGS vectors (pTRV-RNA1/pTRV-RNA2:PDS). Supplementary Table S4. Monthly temperatures in Santa Barbara, CA in 2009. Supplementary Table S5. Monthly average temperatures in Scotland 1971–2000. Supplementary file 2: Supplementary Figures. Supplementary figure S1. Disease symptoms in host plant species mechanically infected with TRV California. Supplementary figure S2. Detection of the TRV infection in host plants. Supplementary Figure S3. N. attenuata plants inoculated with the TRV California vector system, grown at different temperatures. Supplementary figure S4. Systemic silencing of the PDS gene in N. attenuata induced with the TRV California and PpK20 vectors after growth at 28°C and 30°C (sap inoculated). Supplementary figure S5. Swapping of RNA1 and RNA2 vectors of California and PpK20 isolates. Supplementary figure S6. ClustalW analysis of the RNA dependent RNA polymerase (RdR) proteins from the TRV California and TRV PpK20 isolates. Supplementary figure S7. ClustalW analysis of the 16 kDa Suppressor proteins (A), Movement proteins (B) and Coat proteins (C) from the TRV California and TRV PpK20 isolates. Supplementary file 3: Original gel images of Fig. 1D, E, S2A and S2B with legends. Supplementary file 4: Original gel image file (JPEG format) Fig. 1D. Supplementary file 5: Original gel image file (JPEG format) Fig. 1E. Supplementary file 6: Original gel image file (JPEG format) Fig. S2A. Supplementary file 7: Original gel image file (JPEG format) Fig. S2B. [file 12870_2021_3324_MOESM1_ESM.zip › Rahman et al Supplementary tables.docx]

**Supplementary Tables**

**Table S1.** GenBank accessions of full-length RNA1 and RNA2 genomes of different TRV isolates.

| **TRV isolate** | **Accessions of TRV-RNA1** | **Accessions of TRV-RNA2** |
| --- | --- | --- |
| 1AKBH | JX912715 |  |
| AL | HM195288 |  |
| BM |  | AY166663 |
| Cot2 |  | AY166662 |
| Ho | JQ235203 |  |
| K |  | AJ536414 |
| MI-1 | GQ903771 | GQ903772 |
| ORY | AF034622 | AF034621 |
| PaY4 |  | AJ250488 |
| PpK20 | AF166084, AF314165, AF406990 | Z36974 |
| PpO85 | AJ586803 |  |
| South Korea |  | AB369280 |
| SYM | D00155, X06172 |  |
| TCM |  | X03955 |
| TpO1 |  | AJ009833 |
| Rostock |  | AJ272198 |
| TRV-PallM-3 |  | DQ448617 |
| 11r21 | KF758790 |  |
| Deb57 | KF758791 |  |
| Mlo7 | KF758792 |  |
| Slu24 | KF758793 |  |
| Pp085 | AJ586803 |  |
| TRV-PmF-12 |  | DQ448615 |
| OR2 |  | AY166661 |
| Umt1 |  | AY166660 |
| ON |  | Z97357 |

**Table S2**. List of primers used.

| **Purpose** | **Primer name** | **Sequence 5’ to 3’** |
| --- | --- | --- |
| RNA1 sequencing, PCR | TRV21 | GATGTCTGCTGAGCAGAGGAGTC |
| RNA1 sequencing | TRV23 | GCTTATGCGTCGAGACATCCGAG |
| RNA1 PCR, cloning | TRV24 | CCAACTCTAGCTTGGGTCAGCAC |
| RNA1 PCR, sequencing | TRV25 | CGAGAATCTACACTTTGCTTGTTC |
| RNA1 PCR, sequencing | TRV26 | GACTTCAAGTTGAAAGACTTGATG |
| RNA1 sequencing, PCR | TRV27 | CCATCTACTGATATATATTCTTCGG |
| RNA1 cDNA, PCR, cloning | TRV40 | CGTAATAACGCTTACGTAGGCGAG |
| RNA1 sequencing, PCR | TRV41 | CATCACTTTAAAAGTACTTACTCACC |
| RNA1 PCR | TRV42 | ATAAAACATTTCAATCCTTTGAACG |
| RNA1 PCR, cloning | TRV43 | CGCGGTAGAACGTGCTAATTGG |
| RNA1 PCR, cloning, sequencing | TRV44 | CAGAGATTCAAAGCTCGAGCTTG |
| RNA1 cDNA, PCR, cloning, sequencing | TRV45 | GTAGCTGGTCTGTAAGGACCATC |
| RNA1 PCR, cloning | TRV46 | CGGTGGTCGGTTCATCTAGAAGC |
| RNA1 PCR | TRV47 | CCCTCAGAGATACAACCCACCTTCC |
| RNA1 sequencing | TRV49 | CAGCTAATCCTTGCGTCGATGTG |
| RNA1 sequencing | TRV50 | CGCAGAACATTGGGACGTCGTAC |
| RNA1 sequencing | pJET1_2 FOR | CGACTCACTATAGGGAGAGCGGC |
| RNA1 sequencing | pJET1_2 REV | AAGAACATCGATTTTCCATGGCAG |
| RNA2 PCR, cloning | TRV51 | ATAAAACATTGCACCTATGGTGTTGCCC |
| RNA2 cDNA, PCR, cloning | TRV52 | CGTAATAACGCTTACGTAGGCGAGGG |
| pCR-RNA2 sequencing | TRV53 | CATGGGTTGAGGAGAAGAACTGG |
| pCR-RNA2 sequencing | TRV54 | CATCCACAATTACTTCCCACGC |
| pCR-RNA2 sequencing | TRV55 | GCATGGGCATCACGATAGTCCG |
| pCR-RNA2 sequencing | TRV56 | CCGTCCAGATCCACGTAACCACC |
| pCR-RNA2 sequencing | M13 -20 FOR | GTAAAACGACGGCCAG |
| pCR-RNA2 sequencing | M13 REV | CAGGAAACAGCTATGAC |
| pTRV11 construction | TRV57 | GCGGCGATTAATCTGCAGGATATCCAGAGATTCAAAGCTCGAGCTTGCG |
| pTRV11 construction | TRV58 | GCGGCGGGATCCACTAGTGGGCGTAATAACGCTTACGTAGGCGAGGG |
| pTRV12 construction | TRV59 | GCGGCGCTGCAGCATTTAGCCGAGATTTACATCTCACTG |
| pTRV12 construction, pTRV13 construction | TRV60 | GCGGCGCTGTATTCAACCTTGTACTGCGCAACTG |
| pTRV13 construction | TRV61 | GCGGCGCTGCAGGATATCGGGCCCCGGTTCATCTAGAAGCGGACTGGGTTTGC |
| pTRV14 construction | TRV62 | CGACTTCGCAGTCGAACGAATGAGTGATTATGTCATAGTATGCGATCAGACTTATCTTTGCAATAACAGGTAATAATC |
| pTRV14 construction | TRV63 | GCGGCGAGATCTAGGGAACTTAAATTGTCCAAGATCAACCTG |
| pTRV14 construction | TRV64 | GTGACTGGTCGGGATTTTTAGAAGTGGCTGTCTGGGAACGTGGTATGTTGGTCAACGACTTCGCAGTCGAACGAATGAG |
| pTRV14 construction | TRV65 | GCGGCGGGTCACCGAACACGCTTACGTGTTCCAACCAGACAAACGTATGAGTGACTGGTCGGGATTTTTAGAAGTG |
| pTRV15 construction | TRV66 | GCGTCGAGACATCCGAGATGGACTGG |
| pTRV15 construction | TRV67 | GTCTTCCAACAGCAAACCCAGTCCGC |
| pTRV16 construction | TRV68 | GCGGCGCTGCAGATAAAACATTTCAATCCTTTGAACGCGGTAGAACGTGCTAATTGGATTTTG |
| pTRV16 construction | TRV69 | GCGGCACGGATTCTCAACATCTAAGACATG |
| pTRV17 construction | TRV70 | GCGGCGTACGTAAGCGTTATTACGCCCCTCGAGTTTCTCCATAATAATGTGTG |
| pTRV17 construction | TRV71 | GCGGCGACTAGTTAATTCGGGGGATCTGGATTTTAG |
| pTRV-RNA1 construction | TRV72 | GCGGCGGTTTAAACTGAAGGCGGGAAACGACAATCTGATCCGGCGCGCCAAGCTCCACGTGAGCTTCATGGAGTCAAAGATTC |
| pTRV-RNA1 construction | TRV73 | GCGGCGCTGCAGGTCAAGAGTCCCCCGTGTTCTCTC |
| pTRV21 construction | TRV74 | GCGGCGCTGCAGGGGCCCGTCGACGGTACCCCCGGGGAGCTCGGTCACCGGGTTGACGCAGGTGCGTGGGAAG |
| pTRV21 construction | TRV75 | GCGGCGCTCGAGCTTCGTAATAACGCTTACGTAGGCGAGGG |
| pTRV-RNA2 construction | TRV76 | GCGGCGCTGCAGATAAAACATTGCACCTATGGTGTTGC |
| pTRV-RNA2 construction | TRV77 | GCGGCGGTCGACCGTGACCCTCTGAAAGAGTATCTGAC |
| pTRV-RNA2:PDS construction | PDS43 | GCGGCGGGTACCTCAGCTCGATCTTTTTTATTCGTGAG |
| pTRV-RNA2:PDS construction | PDS44 | GCGGCGGAGCTCAGGCACTCAACTTTATAAACCCTGAC |
| pTRV-RNA2:AOC construction, sequencing | AOC7 | GCGGCGGAGCTCCCTGCCTATCTTCGCTTGAG |
| pTRV-RNA2:AOC construction, sequencing | AOC8 | GCGGCGGGTACCCCGGTAACAGCAAGATAAG |
| pTRV-RNA1 sequencing | CalSeq_1-1 | CAAGTTAATGGAAATTTATCCAG |
| pTRV-RNA1 sequencing | CalSeq_1-2 | CTAGAGTGGTTCACGCTGCTATGC |
| pTRV-RNA1 sequencing | CalSeq_1-3 | CATAAATGGGGCGGCTGTGAAG |
| pTRV-RNA1 sequencing | CalSeq_1-4 | GATGTTGAGAATCCGTGCCGC |
| pTRV-RNA1 sequencing | CalSeq_1-5 | CAAACGTGTCGATTACTTCCAAG |
| pTRV-RNA1 sequencing | CalSeq_1-6 | GTATGCGATCAGACTTATCTTTGC |
| pTRV-RNA1 sequencing | CalSeq_1-7 | GATCTAGGACCGGTCGACTGTTC |
| pTRV-RNA1 sequencing | CalSeq_1-8 | CTAATTCAATGACAGTAAGGAAG |
| pTRV-RNA1 sequencing | CalSeq_1-9 | GGCAACGACTGACTGCAACCTG |
| pTRV-RNA1 sequencing | CalSeq_1-10 | CTTCAAAGAAATCAATGAACGC |
| pTRV-RNA1 sequencing | CalSeq_1-11 | CGTCCCAATGTTCTGCGGGAAG |
| pTRV-RNA1 sequencing | CalSeq_1-12 | GAGACTGTCCTAAATCATGTTG |
| pTRV-RNA1 sequencing | CalSeq_1-13 | GATGAAGACGATTGAGGCGAAG |
| pTRV-RNA1 sequencing | CalSeq_LB-1 | CTTGGCGGCGAATGGGCTGACCG |
| pTRV-RNA1 sequencing | CalSeq_P-1 | CTGCAGGTCAAGAGTCCCCCGTG |
| pTRV-RNA1 sequencing | CalSeq_RB-1 | GTATATATGAGTAAACTTGGTCTG |
| pTRV-RNA1 sequencing, pTRV-RNA2 sequencing | CalSeq_P-2 | CGCACAATCCCACTATCCTTCGC |
| pTRV-RNA1 sequencing, pTRV-RNA2 sequencing | CalSeq_T-1 | GATGTTCCTTTCGGAATTGATCAG |
| pTRV-RNA2 sequencing | CalSeq_2-1 | CTGGAAGAAGCATAATTATACTG |
| pTRV-RNA2 sequencing, pTRV-RNA2:PDS sequencing | CalSeq_2-2 | CCACCAAGGTTGGACGACTCGTC |
| pTRV-RNA2 sequencing | CalSeq_2-3 | GGTCACCGGGTTGACGCAGGTGC |
| qPCR AOC gene | AOC_3F_24 | CAATCTCTTAAACTTAGCACCTCC |
| qPCR AOC gene | AOC_4R_23 | GTTGTTGAACTTTAGTGGCTGTG |
| qPCR EF1 gene | EFv7-F | TGGTATGGTTAAGATGCTTCCC |
| qPCR EF1 gene | EFv7-R | TGTCAACGCTCTTGATAACAC |
| qPCR PDS gene | Na_PDS_F | GCTCTTCAGCAGAAGTCCAC |
| qPCR PDS gene | Na_PDS_R | CAGGTGCAAATACCAATTCCA |

**Table S3.** Effect of different growth temperatures on *PDS* gene silencing in *N. attenuata* induced by TRV-California VIGS vectors (pTRV-RNA1/pTRV-RNA2:PDS).

| Growth temperature | Number of *PDS* bleached plants ***** | | | | Mean of silencing | Efficiency of silencing  (%) |
| --- | --- | --- | --- | --- | --- | --- |
|  | Experiment 1 | Experiment 2 | | Experiment 3 |  |  |
| 22°C | 6/10 | 8/10 | | 7/10 | 7.0±1.0 | 70 |
| 26°C | 7/10 | 8/10 | | 8/10 | 7.7± 0.57 | 77 |
| 28°C | 7/10 | 8/10 | | 7/10 | 7.3±0.57 | 73 |
| 30°C | 4/10 | | 3/10 | 5/10 | 3.2± 1.0 | 40 |

* In each experiment 10 plants were inoculated.

**Table S4.** Monthly temperatures in Santa Barbara, CA in 2009.

| Month | Highest max  temperature (°C) | Lowest min  temperature (°C) | Average  Temperature (°C) |
| --- | --- | --- | --- |
| January | Not available | Not available | 13.1 |
| February | 27.2 | 2.2 | 13.8 |
| March | 27.8 | 4.4 | 14.95 |
| April | 36.7 | Not available | 16.0 |
| May | 36.1 | 8.9 | 18.4 |
| June | 28.3 | 12.8 | 19.5 |
| July | 33.3 | 12.8 | 21.3 |
| August | 35.6 | 12.2 | 21.3 |
| September | 32.8 | 13.9 | 22.5 |
| October | 38.9 | Not available | 18.1 |
| November | 36.1 | Not available | 14.95 |
| December | Not available | Not available | 13.2 |
| Source: U.S. Climate data ([www.usclimatedata.com)](http://www.usclimatedata.com)) | | | |

**Table S5.** Monthly average temperatures in Scotland 1971 - 2000.

| Month | Temperature °C  (Max) | Temperature °C  (Min) |
| --- | --- | --- |
| January | 5 | 0.2 |
| February | 5.2 | 0.1 |
| March | 6.9 | 0.9 |
| April | 9.3 | 2.1 |
| May | 12 | 4.5 |
| June | 12.8 | 7.2 |
| July | 14.9 | 9.3 |
| August | 16.6 | 9.2 |
| September | 13.9 | 7.2 |
| October | 10.8 | 4.9 |
| November | 7.4 | 2.0 |
| December | 5.7 | 4.0 |
| Year | 10.5 | 4.0 |
| Source: Met Office, UK, 2001. ["Scotland 1971–2000 averages"](https://web.archive.org/web/20040430030137/http://www.metoffice.gov.uk/climate/uk/averages/19712000/areal/scotland.html) (<https://web.archive.org/web/20040430030137/http://www.metoffice.gov.uk/climate/uk/averages/19712000/areal/scotland.html)> | | |
