# Supplementary figures and images for: California TRV-based VIGS vectors mediate gene silencing at elevated temperatures but with greater growth stunting
Source: BMC Plant Biol. 2021 Nov 22;21:553. doi: 10.1186/s12870-021-03324-8 (PMC8607596; doi:10.1186/s12870-021-03324-8)

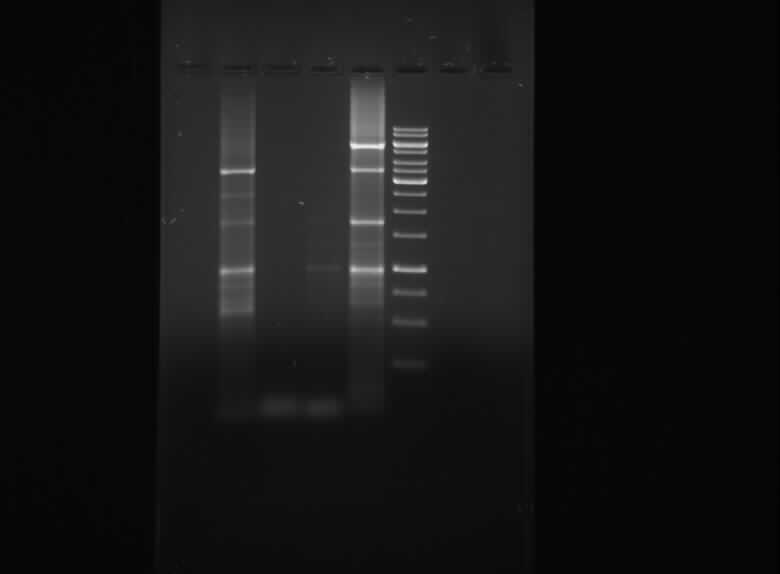

Supplement: Supplementary file 1 — Additional file 1: Supplementary Table S1. GenBank accessions of full-length RNA1 and RNA2 genomes of different TRV isolates. Supplementary Table S2. List of primers used. Supplementary Table S3. Effect of different growth temperatures on PDS gene silencing in N. attenuata induced by TRV California VIGS vectors (pTRV-RNA1/pTRV-RNA2:PDS). Supplementary Table S4. Monthly temperatures in Santa Barbara, CA in 2009. Supplementary Table S5. Monthly average temperatures in Scotland 1971–2000. Supplementary file 2: Supplementary Figures. Supplementary figure S1. Disease symptoms in host plant species mechanically infected with TRV California. Supplementary figure S2. Detection of the TRV infection in host plants. Supplementary Figure S3. N. attenuata plants inoculated with the TRV California vector system, grown at different temperatures. Supplementary figure S4. Systemic silencing of the PDS gene in N. attenuata induced with the TRV California and PpK20 vectors after growth at 28°C and 30°C (sap inoculated). Supplementary figure S5. Swapping of RNA1 and RNA2 vectors of California and PpK20 isolates. Supplementary figure S6. ClustalW analysis of the RNA dependent RNA polymerase (RdR) proteins from the TRV California and TRV PpK20 isolates. Supplementary figure S7. ClustalW analysis of the 16 kDa Suppressor proteins (A), Movement proteins (B) and Coat proteins (C) from the TRV California and TRV PpK20 isolates. Supplementary file 3: Original gel images of Fig. 1D, E, S2A and S2B with legends. Supplementary file 4: Original gel image file (JPEG format) Fig. 1D. Supplementary file 5: Original gel image file (JPEG format) Fig. 1E. Supplementary file 6: Original gel image file (JPEG format) Fig. S2A. Supplementary file 7: Original gel image file (JPEG format) Fig. S2B. [file 12870_2021_3324_MOESM1_ESM.zip › original_file_gel_1D.jpg]

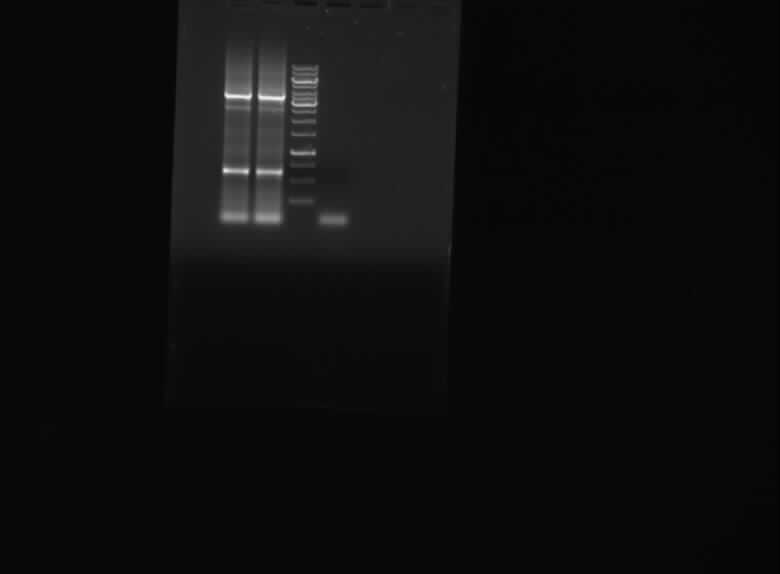

Supplement: Supplementary file 1 — Additional file 1: Supplementary Table S1. GenBank accessions of full-length RNA1 and RNA2 genomes of different TRV isolates. Supplementary Table S2. List of primers used. Supplementary Table S3. Effect of different growth temperatures on PDS gene silencing in N. attenuata induced by TRV California VIGS vectors (pTRV-RNA1/pTRV-RNA2:PDS). Supplementary Table S4. Monthly temperatures in Santa Barbara, CA in 2009. Supplementary Table S5. Monthly average temperatures in Scotland 1971–2000. Supplementary file 2: Supplementary Figures. Supplementary figure S1. Disease symptoms in host plant species mechanically infected with TRV California. Supplementary figure S2. Detection of the TRV infection in host plants. Supplementary Figure S3. N. attenuata plants inoculated with the TRV California vector system, grown at different temperatures. Supplementary figure S4. Systemic silencing of the PDS gene in N. attenuata induced with the TRV California and PpK20 vectors after growth at 28°C and 30°C (sap inoculated). Supplementary figure S5. Swapping of RNA1 and RNA2 vectors of California and PpK20 isolates. Supplementary figure S6. ClustalW analysis of the RNA dependent RNA polymerase (RdR) proteins from the TRV California and TRV PpK20 isolates. Supplementary figure S7. ClustalW analysis of the 16 kDa Suppressor proteins (A), Movement proteins (B) and Coat proteins (C) from the TRV California and TRV PpK20 isolates. Supplementary file 3: Original gel images of Fig. 1D, E, S2A and S2B with legends. Supplementary file 4: Original gel image file (JPEG format) Fig. 1D. Supplementary file 5: Original gel image file (JPEG format) Fig. 1E. Supplementary file 6: Original gel image file (JPEG format) Fig. S2A. Supplementary file 7: Original gel image file (JPEG format) Fig. S2B. [file 12870_2021_3324_MOESM1_ESM.zip › original_file_gel_1E.jpg]

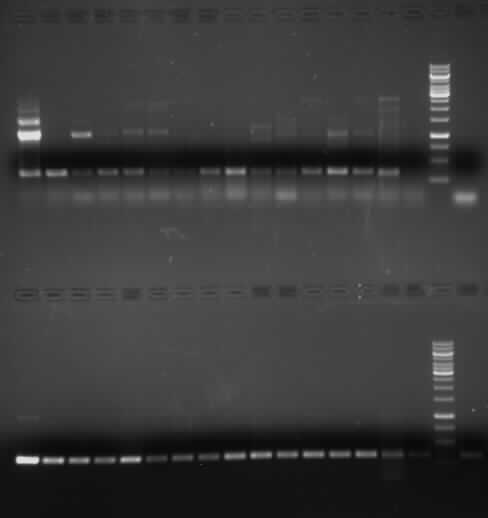

Supplement: Supplementary file 1 — Additional file 1: Supplementary Table S1. GenBank accessions of full-length RNA1 and RNA2 genomes of different TRV isolates. Supplementary Table S2. List of primers used. Supplementary Table S3. Effect of different growth temperatures on PDS gene silencing in N. attenuata induced by TRV California VIGS vectors (pTRV-RNA1/pTRV-RNA2:PDS). Supplementary Table S4. Monthly temperatures in Santa Barbara, CA in 2009. Supplementary Table S5. Monthly average temperatures in Scotland 1971–2000. Supplementary file 2: Supplementary Figures. Supplementary figure S1. Disease symptoms in host plant species mechanically infected with TRV California. Supplementary figure S2. Detection of the TRV infection in host plants. Supplementary Figure S3. N. attenuata plants inoculated with the TRV California vector system, grown at different temperatures. Supplementary figure S4. Systemic silencing of the PDS gene in N. attenuata induced with the TRV California and PpK20 vectors after growth at 28°C and 30°C (sap inoculated). Supplementary figure S5. Swapping of RNA1 and RNA2 vectors of California and PpK20 isolates. Supplementary figure S6. ClustalW analysis of the RNA dependent RNA polymerase (RdR) proteins from the TRV California and TRV PpK20 isolates. Supplementary figure S7. ClustalW analysis of the 16 kDa Suppressor proteins (A), Movement proteins (B) and Coat proteins (C) from the TRV California and TRV PpK20 isolates. Supplementary file 3: Original gel images of Fig. 1D, E, S2A and S2B with legends. Supplementary file 4: Original gel image file (JPEG format) Fig. 1D. Supplementary file 5: Original gel image file (JPEG format) Fig. 1E. Supplementary file 6: Original gel image file (JPEG format) Fig. S2A. Supplementary file 7: Original gel image file (JPEG format) Fig. S2B. [file 12870_2021_3324_MOESM1_ESM.zip › original_file_gel_S2A.jpg]

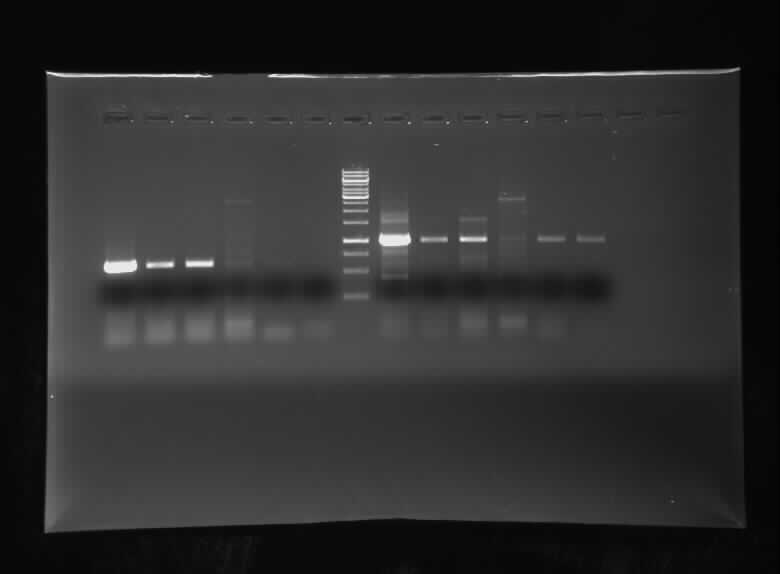

Supplement: Supplementary file 1 — Additional file 1: Supplementary Table S1. GenBank accessions of full-length RNA1 and RNA2 genomes of different TRV isolates. Supplementary Table S2. List of primers used. Supplementary Table S3. Effect of different growth temperatures on PDS gene silencing in N. attenuata induced by TRV California VIGS vectors (pTRV-RNA1/pTRV-RNA2:PDS). Supplementary Table S4. Monthly temperatures in Santa Barbara, CA in 2009. Supplementary Table S5. Monthly average temperatures in Scotland 1971–2000. Supplementary file 2: Supplementary Figures. Supplementary figure S1. Disease symptoms in host plant species mechanically infected with TRV California. Supplementary figure S2. Detection of the TRV infection in host plants. Supplementary Figure S3. N. attenuata plants inoculated with the TRV California vector system, grown at different temperatures. Supplementary figure S4. Systemic silencing of the PDS gene in N. attenuata induced with the TRV California and PpK20 vectors after growth at 28°C and 30°C (sap inoculated). Supplementary figure S5. Swapping of RNA1 and RNA2 vectors of California and PpK20 isolates. Supplementary figure S6. ClustalW analysis of the RNA dependent RNA polymerase (RdR) proteins from the TRV California and TRV PpK20 isolates. Supplementary figure S7. ClustalW analysis of the 16 kDa Suppressor proteins (A), Movement proteins (B) and Coat proteins (C) from the TRV California and TRV PpK20 isolates. Supplementary file 3: Original gel images of Fig. 1D, E, S2A and S2B with legends. Supplementary file 4: Original gel image file (JPEG format) Fig. 1D. Supplementary file 5: Original gel image file (JPEG format) Fig. 1E. Supplementary file 6: Original gel image file (JPEG format) Fig. S2A. Supplementary file 7: Original gel image file (JPEG format) Fig. S2B. [file 12870_2021_3324_MOESM1_ESM.zip › original_file_gel_S2B.jpg]
